# iTRAQ-based proteomic study on monocyte cell model discovered an association of LAMP2 downregulation with HIV-1 latency

**DOI:** 10.1186/s12953-024-00230-3

**Published:** 2024-05-15

**Authors:** Lin Yin, Qimin Wang, Siyuan Liu, Jun Chen, Yujiao Zhang, Lingqing Lu, Hongzhou Lu, Zhigang Song, Lijun Zhang

**Affiliations:** 1 Shanghai Public Health Clinical Center, Fudan University, Shanghai, 201508 China; 2 Shanghai Ninth People’s Hospital, Shanghai Jiaotong University School of Medicine, Shanghai, 200011 China; 3 Department of Infectious Diseases, National Clinical Research Center for Infectious Diseases, The Third People’s Hospital of Shenzhen, Shenzhen, 518112 China

**Keywords:** Proteomics, Plasma membrane, HIV latency, iTRAQ, LAMP2

## Abstract

**Background:**

Patients with immunodeficiency virus-1 (HIV-1) infection are challenging to be cured completely due to the existence of HIV-1 latency reservoirs. However, the knowledge of the mechanisms and biomarkers associated with HIV-1 latency is limited. Therefore, identifying proteins related to HIV-1 latency could provide new insights into the underlying mechanisms of HIV-1 latency, and ultimately contribute to the eradication of HIV reservoirs.

**Methods:**

An Isobaric Tags for Relative and Absolute Quantification (iTRAQ)-labeled subcellular proteomic study was performed on an HIV-1 latently infected cell model (U1, a HIV-1-integrated U937 cell line) and its control (U937). Differentially expressed proteins (DEPs) were analyzed using STRING-DB. Selected DEPs were further evaluated by western blotting and multiple reaction monitoring technology in both cell model and patient-derived cluster of differentiation 4 (CD4)^+^ T cells. Finally, we investigated the relationship between a specific DEP lysosome-associated membrane glycoprotein 2 (LAMP2) and HIV-1 reactivation by panobinostat or lysosome regulation by a lysosomotropic agent hydroxychloroquine in U1 and U937 cells.

**Results:**

In total, 110 DEPs were identified in U1 cells comparing to U937 control cells. Bioinformatics analysis suggested associations of the altered proteins with the immune response and endosomal/lysosomal pathway. LAMP2, leukocyte surface antigen CD47, CD55, and ITGA6 were downregulated in HIV-1 latent cells. Downregulated LAMP2 was further confirmed in resting CD4^+^ T cells from patients with latent HIV-1 infection. Furthermore, both HIV-1 reactivation by panobinostat and stimulation with hydroxychloroquine upregulated LAMP2 expression.

**Conclusions:**

Our results indicated the involvement of the endosomal/lysosomal pathway in HIV-1 latency in macrophage cell model. The down-modulation of LAMP2 was associated with HIV latency, and the restoration of LAMP2 expression accompanied the transition of viral latency to active infection. This study provides new insights into the mechanism of HIV-1 latency and potential strategies for eradicating HIV-1 reservoirs by targeting LAMP2 expression.

**Supplementary Information:**

The online version contains supplementary material available at 10.1186/s12953-024-00230-3.

## Background

Although antiretroviral therapy (ART) drastically increases the survival of individuals infected with immunodeficiency virus-1 (HIV-1) [1], patients still need to take drugs during their lifetime because the virus cannot be fully eradicated from their reservoirs [2, 3]. In an HIV-1 reservoir, integrated HIV-1 is not affected by the existing ART unless viral transcription is activated [4] and cannot be recognized by the immune system [5]. Therefore, it is very important to perform studies focusing on HIV-1 reservoirs. Although many strategies were developed to eliminate latent HIV-1, including the “Shock and Kill” strategy, gene therapy and/or gene editing, and stem cell transplantation [6], the latent reservoir still represents the main barrier to eradicating HIV from infected individuals. Two of main reasons are that the mechanism of HIV-1 latency has not been completely elucidated [7]. Additionally, the biomarkers of HIV-1 latent cells were very limited [8]. Therefore, it is very important to discover proteins related to HIV-1 latency, which could broaden our knowledge about underlying mechanisms of HIV-1 latency.

To identify the proteins related to HIV-1 latency, the first step is selecting an appropriate cell model. So far, the best-characterized cellular reservoirs of HIV are resting CD4^+^ T cells [9], which are widely used in HIV research. However, latently infected CD4^+^ T cells are rare (1–100 per million CD4^+^ T cells) in patients with viral suppression. Therefore, in vivo identification of cellular biomarkers is seriously impeded [10]. Furthermore, the cells of HIV-1-latent reservoirs are unexpectedly dynamic and diverse [11], including CD4^+^ T cells, macrophages, and monocytes. Moreover, HIV-1 latency in macrophages [12] differs from that in CD4^+^ T cells [13]. Therefore, there is no suitable model to completely simulate HIV-1 latency. Several latency models have been developed, including transformed lymphocytic cell lines and resting CD4^+^ T cells. For example, cell lines such as U1 and J1.1 cells, exhibiting the characteristics of HIV-1 latency, have been developed and used for biomarker discovery [14, 15].

Since latently infected cells do not express viral antigens and are not detected or cleared by the immune system, proteins specifically expressed on the cellular surface might offer new clues for eradicating HIV-1 because these proteins play a crucial role in HIV-1 entry, budding, and immune recognition. For example, cell surface proteins such as CD4, CXCR4, and CCR5 serve as receptors or co-receptors for active HIV-1 entry [15, 16]. Additionally, CD44 is upregulated in reactivated HIV-latent cells (J1.1 and U1) [14]. Therefore, identifying proteins on the cellular surface of latent HIV-1-infected cells is crucial for understanding the virus and developing strategies for its eradication. However, identifying proteins within the plasma membrane (PM) is a big challenge due to their low abundance and high hydrophobicity [17]. So far, various subcellular proteomic methods, including two-dimensional electrophoresis and stable isotope labeling by amino acids in cell culture, have been developed to identify PM proteins [18, 19], including in HIV-1 latently infected cells [8, 19]. Some proteins related to HIV-1 latency have been discovered, such as CD147, CD231 [8], the X-linked inhibitor of apoptosis, phosphate of Bruton’s tyrosine kinase [20], and CD30 [21]. However, it is still far from understanding latent HIV-1 infected cells. Therefore, it is necessary to identify potential biomarkers using new technology. Isobaric Tags for Relative and Absolute Quantification (iTRAQ)-based proteomic technology is one of high-throughput screening technologies that has been widely used in recent years. iTRAQ technology has the advantages of high labeling efficiency, high sensitivity, wide-ranging applications, and the ability to compare the protein expression levels of up to eight samples simultaneously [22].

To identify the proteins in cellular surface related to HIV-1 latency, and broaden our knowledge about underlying mechanisms of HIV-1 reservoirs, we performed a subcellular proteomics study in this study. The PM from an HIV-1-latent cell model was enriched, and iTRAQ-based proteomic technology was used to quantify proteins in cell lines, including a latent HIV-1 infected cell (U1) and its control cell (U937). Differentially expressed proteins (DEPs) were confirmed in cell lines (U1 vs. U937, J-Lat (Jurkat cells latently infected with HIV-1) vs. Jurkat) and in primary resting CD4^+^ T cells collected from patients with HIV-1 infection. The relationship between a DEP (lysosome-associated membrane glycoprotein 2 (LAMP2)) and HIV-1 reactivation (using panobinostat) or lysosome regulation (using the lysosomotropic agent hydroxychloroquine) was studied.

## Materials and methods

### Cell lines

U1 (U937 cells latently infected with HIV-1) [14] and J-Lat (Jurkat cells latently infected with HIV-1) were generously provided by Shibo Jiang (Fudan University, Shanghai, China) and Jianqing Xu (Fudan University, Shanghai, China), respectively. U1, U937, J-Lat, and Jurkat cells were cultured in RPMI 1640 with 10% fetal bovine serum, 100 U/mL penicillin, and 100 mg/mL streptomycin (Thermo Fisher Scientific, Shanghai, China), under standard cell culture conditions. U1 and U937 cells were utilized for PM enrichment, flow cytometry detection, and experiments involved HIV-1 reactivation and lysosomotropic stimulation. The PM-enriched samples from U1 and U937 cells were further used for proteomic study and western blotting. In contrast, J-Lat and Jurkat cells were exclusively employed for PM enrichment, followed by western blot analysis.

### Cell models for HIV-1 reactivation and lysosomotropic stimulation

In order to assess the effects of HIV-1 reactivation on LAMP2 protein expression, concentration- and time-dependent experiments were performed using panobinostat (404950-80-7, MedChemExpress LLC, Neaw Zealand, USA) (an HIV-1 reactivation agent [23]). Briefly, U1 and U937 cells (5 × 10^5^) were cultured in 12-well plates with 2 mL culture medium. Cells were exposed to panobinostat at a final concentration of 0, 10, or 25 nM for 24 h, or 25 nM for 0, 24, or 48 h. Cell supernatants were collected for HIV-1 p24 detection, while cells were collected for protein extraction with RIPA cell lysis buffer, followed by western blot detection of LAMP2 expression. Each group was seeded in three replicate wells, and the experiment was repeated twice.

Furthermore, to assess the relationship between LAMP2 expression and lysosome regulation, the cells were exposed to hydroxychloroquine (Laboratory of the Government Chemist, Luckenwalde, Germany) (a lysosomotropic agent) [24] at a final concentration of 0, 10, or 25 µM for 24 h, or 10 µM for 0, 24, or 48 h. The following detections were the same as those in panobinostat treatment, with supernatants for HIV-1 p24 detection and cells for western blot detection of LAMP2 expression.

### Volunteer enrollment and sample collection

This study was reviewed and approved by the Ethics Committee of Shanghai Public Health Clinical Center (approval no.: 2017-Y037). Informed consents were obtained from all participants. Three groups of volunteers were enrolled, including: (1) latent HIV-1 infected patients [25] (*n* = 5) who had been treated with ART for at least six months and maintained a viral load of < 50 copies/mL [19]; (2) untreated HIV-1 infected patients (*n* = 5) who had not received ART and had a virus load exceeding 10^5^ copies/mL; and (3) healthy volunteers without HIV-1 (*n* = 5). All participants were aged 18–65 years and had no history of cancer, pregnancy, breastfeeding, hepatitis B virus (HBV), HCV, and tuberculosis infection. Peripheral blood (5 mL) was collected into a K_2_EDTA anticoagulated tube and immediately used for the separation of resting CD4^+^ T cell.

### Resting CD4^+^ T cell separation

Resting CD4^+^ T cells were enriched as described in our previous study [19]. Briefly, peripheral blood mononuclear cells (PBMC) were separated from blood using a Ficoll density centrifugation. Subsequently, CD4^+^ T cells were isolated by negative selection through incubating PBMC with anti-CD25/CD69/HLA-DR coated beads packed in a CD4^+^ T cell isolation kit (130-096-533, Miltenyi Biotec, Germany). The eluted fragments were collected as resting CD4^+^ T cells, which were then detected using a flow cytometry (Beckman, Florida, USA). These cells were stored at -80℃ for the validation of LAMP2 expression by mass spectrometry-based multiple reaction monitoring (MRM).

### PM enrichment

The PM was enriched using a PM protein extraction kit (ab65400, Abcam, Cambridge, Massachusetts, USA) following the manufacturer’s protocol and a previous study [19]. Briefly, suspended U1, U937, J-Lat, and Jurkat cells (5–8 × 10^8^ cells/each) were centrifuged at 500 g for 5 min to collect the cell pellet. The pellet was washed twice with phosphate buffered solution (PBS) and homogenized in PBS with protease inhibitors. A portion of the homogenate, referred to as the ‘homogenate of whole-cell’, was collected, and used for protein extraction. The rest homogenates were continually centrifuged at 700 *g* to remove the nuclear and unbroken cells. The resulting supernatant was then centrifuged at 10,000 g for 30 min. The resulting pellet, representing the total membrane (TM), was collected and resuspended in 200 µL of the upper phase solution. This suspension was combined with 200 µL of the lower phase solution, mixed thoroughly, incubated on ice for 5 min, and centrifuged at 1000 *g* for 5 min at 4 °C to collect the upper phase, which was referred to as the PM fraction (PM). The homogenate of whole-cell, TM, and PM were used for protein extraction, followed by western blot for membrane purification detection, proteomic study, and DEP evaluation. To ensure an adequate amount of protein samples and make biological replicates, the PM enrichment process was triplicated and used for subsequent experiments, including protein extraction, western blot, digestion, and labeling of peptides.

### Protein extraction

Proteins were extracted from the whole-cell homogenate, TM or PM with a triple volume of lysis buffer (nonidet P 40 (1%), urea (8 M), thiourea (2 M), 3-[(3-cholamidopropyl)-dimethylammonio]-1-propane sulfonate (4%), phenylmethanesulfonyl fluoride (0.5 mM), and dithiothreitol (DTT) (65 mM)). After incubating for three hours at room temperature, the samples were centrifuged to remove any insoluble fraction, and the resulting supernatants containing the total extractable protein were collected. Total protein concentrations were quantified using the Bradford method and stored at -80℃ for further usage.

For U1 and U937 cells undergoing HIV-1 reactivation and lysosomotropic stimulation, as well as CD4^+^ T cells from volunteers, proteins were extracted with RIPA cell lysis buffer, quantified using the bicinchoninic acid method, and used for western blot validation of LAMP2 expression.

### Protein reduction, alkylation and digestion

Based on the measured protein concentrations, various samples from the PM of U1 and U937 cells were diluted to the same concentration and volume. Subsequently, equal amounts of protein (100 µg) from each sample were reduced with DTT to a final concentration of 5 mM for 60 min at 55 °C. After reduction, the samples were alkylated with iodoacetamide at a final concentration of 10 mM for 30 min at room temperature in a dark place, and desalted with a six-fold volume of pre-cooled acetone to precipitate the proteins at − 20 °C overnight. Following precipitation, the samples were centrifuged at 8,000 g for 10 min at 4 °C to collect the precipitate. After volatilizing acetone, the precipitated protein sample was digested with 50 µL of the tryptic solution (triethylammonium bicarbonate (TEAB) with a trypsin concentration of 50 ng/µL) at 37 °C for 16 h. Then, the digested peptide solution was centrifuged at 10,000 g for 10 min to collect the supernatant. The precipitates were washed with 67% acetonitrile (ACN) containing 5% formic acid (FA), and centrifuged to collect the supernatant. Both supernatants were combined, lyophilized, and stored at -80℃ for iTRAQ labeling.

### iTRAQ label

The digested peptide samples from the PM of U1 and U937 cells were re-dissolved in 50 µL of 100 mM TEAB and labeled with eight-channel iTRAQ reagent (AB SCIEX, Massachusetts, USA), as following: three individual samples from U937 were labeled with reagent 117, 118, or 119, and individual U1 samples were labeled with 114, 115, or 116. Furthermore, one-sixth of the above samples was mixed, labeled with 121, and used as a reference sample. The labeling was performed according to the manufacturer’s instructions. Briefly, iTRAQ reagent was dissolved in 150 µL isopropanol at room temperature and added to 50 µL of the digested peptide sample (from 100 µg of proteins). The labeling reaction was performed at room temperature for 2 h. To terminate the labeling reaction, 100 µL of water was added to each sample and incubated for 30 min at room temperature. Subsequently, the seven labeled peptide samples (three from U937, three from U1, and one mixed reference) were mixed, lyophilized, and stored at -80 °C for liquid chromatography-mass spectrometry detection.

### Liquid chromatography-mass spectrometry (LC-MS)

The labeled peptides were dissolved in 110 µL of mobile phases A (2% ACN in water, pH10, ammonium hydroxide (NH_4_OH)) and separated into ten fractions using an Agilent 1100 HPLC System (Agilent, California, USA). Briefly, the labeled peptides were separated at a flow of 300 µL/min using an Agilent Zorbax Extend reverse phase column (5 μm, 150 mm × 2.1 mm) with a solvent gradient as follows: of 0–8 min, 2% B (98% ACN in water, pH10, NH_4_OH); 8–8.01 min, 2–5% B; 8.01–30 min, 5–25% B; 30–40 min, 25–40% B; 40–45 min, 40–90% B; 45–50 min, 90% B; 50–55 min, 90–2% B; and 55–60 min, 2% B. The eluents from 8 to 60 min were collected into 10 centrifugal tubes with one tube collected every minute in a circular manner until the end of the gradient. The separated peptides were lyophilized for mass spectrometry detection.

The dried fractions (10 in total) were separately solubilized in 100 µL of nano-RPLC buffer A (2% ACN containing 0.1% FA), and each underwent LC-MS detection twice, named replicate 1 and replicate 2. In each replicate, 2 µL from each fraction was injected into an online nano-RPLC (Eksigent nano LC-Ultra™ 2D System, AB SCIEX, Massachusetts, USA). Initially, the labeled peptides were desalted by loading onto a C18 nano LC trap column (100 μm × 3 cm, C18, 3 μm, 150 Å) and washed with nano-RPLC buffer A at a flow rate of 2 µL/min for 10 min. Subsequently, the trap was switched to an in-line analytical ChromXP C18 column (75 μm × 15 cm, C18, 3 μm, 120 Å (ChromXP Eksigent, AB SCIEX)), with a flow rate of 300 nL/min. The linear gradient was applied over 65 min (0–45 min, 2–28% B (0.1% FA in ACN); 45–55 min, 28–42% B; 55–60 min, 42–90%B; and 60–65 min, 90% B).

Data acquisition was performed using a Triple TOF 5600 System (AB SCIEX) equipped with a Nanospray III source (AB SCIEX) and a pulled quartz tip as the emitter (New Objectives, USA). The acquisition parameters included an ion spray voltage of 2.5 kV, curtain gas at 30 PSI, nebulizer gas at 5 PSI, and an interface heater temperature of 150 °C. A TOF MS survey scan was performed at m/z 450 − 1500 within 250 ms. The Q1 isolation window was set to unit resolution of 1.0 m/z. For MS2 spectra acquirement, an information -dependent acquisition (IDA) model was used. The top 35 abundant precursor ions (with m/z of 450–1250, charges between + 2 and + 5, and intensity above 300 cps) from each MS1 scan were selected for MS/MS product ion scan (m/z,100 to 1500, 60 ms). The rolling collision energy (CE) was optimized for fragmentation of precursor ions with different charges and molecular weight. The exclusion time was set at 18s, and the total MS and MS2 cycle time was 3s.

The raw data have been deposited to the ProteomeXchange Consortium (http://proteomecentral.proteomexchange.org) via the iProX partner repository with the dataset identifier PXD037308, or iProX with the number of IPX0005164000 (https://www.iprox.cn/page/project.html).

### Protein identification and quantification

The MS/MS data files from all fractions were combined and searched against Swiss-Prot human database (uniprot-human-2.fasta) by ProteinPilot™ software 5.0 (AB SCIEX, USA) with the Paragon algorithm (5.0.0.0, 4767). The search parameters were specified as follows: digestion, trypsin; one missed enzymatic cleavage site; the mass tolerance of ± 0.05, and ± 0.1 Da for precursor ions and fragment ions, respectively. Carbamidomethyl of cysteine, and iTRAQ 8-plex of lysine and the N-terminus were specified as fixed modifications, and oxidation of methionine and iTRAQ 8-plex of tyrosine were specified as variable modifications. Data was normalized for loading error by background correction and bias corrections calculated using ProteinPilot. All identified proteins had a global false discovery rate (FDR) less than 0.01. Protein groups considered for quantification required at least one peptide; and quantification was performed with unique peptides as well as shared peptides. The peak area ratios of the iTRAQ reporter ions of 114:121, 115:121, 116:121, 117:121, 118:121, and 119:121 were used for DEP analysis.

### LAMP2 validation using mass spectrometry-based MRM technology

CD4^+^ T cell proteins from the three groups were extracted and digested as previously described. Before loading onto LC-MS, the peptides were desalted using C18 ZipTips microcolumns (Millipore, USA) and eluted with 50% ACN containing 0.1% FA according to the previously described [26].

The peptides selected for MRM quantification adhered to the following criteria: 1) detection in iTRAQ-based proteomic study; 2) unique peptides reported by MS/MS data searching software; 3) confidence level exceeding 95%; and 4) inclusion as reference peptides for LAMP2 in the proteomics database (https://www.proteomicsdb.org). Ultimately, the peptide sequence of IPLNDLFR was chosen. This peptide (5 mg with purity of 95.19%) and the internal standard peptide of GYSFTTTAER (5 mg with purity of 96.0%) from β-actin were synthesized by Shanghai Qiangyao Biotechnology Co., Ltd. (Shanghai, China).

The synthesized peptide was dissolved in water containing 0.1% FA to the final concentrations of 5–200 ng/mL, and a volume of 10 µL was injected and quantified using ultra-high-performance liquid chromatography/tandem mass spectrometry. Chromatographic separation was conducted on a C18 column (Venusil ASB C18, 2.1 × 50 mm, 5 μm) (Agela Technologies, Tianjin, China) in a Waters ACQUITY UPLC system (Waters company, Massachusetts, USA). The compounds were eluted with 0.1% FA in water (A) and 0.1% FA in ACN (B) at 0.35 mL/min for 3.5 min at a gradient of 0–0.8 min, 2% B; 1.2–2.2 min, 80% B; and 2.5–3.5 min, 2% B. The peptides were detected using A QTRAP 5500 tandem mass spectrometer (MS) (Applied Biosystems/AB SCIEX, Boston, USA) in a positive MRM mode. The MS parameters were optimized to detect the MRM precursors/fragment ions of 494.6 ((M + 2 H)^2+^)/664.2 and 567.1(M + H_2_O + 2 H)^2+^)/912.5 for IPLNDLFR from LAMP2, and GYSFTTTAER from β-actin, respectively. The peptide from β-actin was used as internal standard (IS). Quantification analysis was performed by Analyst 1.6.3 software using the peak area of analysts to that of the IS. Part of the method validation experiments, such as specificity, calibration curves, and accuracy, were performed according to the guidelines of the Food and Drug Administration (FDA) before the relative quantitation of LAMP2 in the CD4^+^ T cells from the volunteers. In this study, five samples from each group were involved, and each sample were detected twice by LC-MS.

### Bioinformatics analysis

R package software (version 4.0.2) was used to analyze the volcano plot, venn diagram, and clustering data [27]. The protein accessions of a total of 110 DEPs were input for bioinformatics analysis through STRING software (Version:11.0b) (https://string-db.org). The up-regulated and down-regulated proteins were submitted separately. Biological process (BP), molecular function (MF), cellular component (CC), Kyoto Encyclopedia of Genes and Genomes (KEGG) pathway, and protein-protein interactions were analyzed by setting a minimum required interaction score of 0.7 (indicating high confidence) and a FDR less than 0.05. When the number of description items for BP, MF, CC, and KEGG pathway (reported by the STRING software) exceeded 10, only the top 10 items were extracted. Interactions between DEPs and HIV proteins were examined using the HIV interaction database (https://www.ncbi.nlm.nih.gov/genome/viruses/retroviruses/hiv-1/interactions). The protein-protein interaction network was constructed using Cytoscape software (version 3.7.1), and node proteins were exported from the STRING software.

### Western blotting

For membrane purification detection, U1 and U937 cells were used. In the analysis of differential protein expression involving LAMP2, CD55, ITGA6, and CD47, U1, U937, J-Lat, and Jurkat cells were employed. The protein fractions analyzed included homogenate, TM, and PM. LAMP2 protein levels were further investigated in U1 and U937 cell homogenate treated with panobinostat or hydroxychloroquine.

Proteins (20 µg) from homogenate, TM, and PM were separated by electrophoresis in a sodium dodecyl sulfate (SDS)-12.5% polyacrylamide gel and transferred to a PVDF membrane (Millipore company, Boston, Massachusetts, USA). After blocking in 10% defatted milk for 2 h at room temperature or overnight at 4℃, blots were incubated for 2 h at 37˚C or overnight at 4℃ with specific primary antibodies (Na^+^/K^+^-ATPase (ab7671, 1:5000), prohibitin (ab28172, 1:2000), LAMP2 (ab199946, 1:1000), CD55 (ab133684, 1:1000), ITGA6 (ab181551, 1:1000), CD47 (ab218810, 1:1000), and β-actin (60009-1-Ig, 1:5000, PROTEINTECH). After three washes with TBST (TBS + Tween), blots were incubated for one hour at room temperature with secondary antibodies (goat anti-mouse IgG-HRP (1:2000, Santa Cruz Biotechnology, California, USA) or goat anti-rabbit IgG-HRP (1:2000, Santa Cruz Biotechnology, California, USA)). After further washes, the immune complexes were revealed by enhanced chemiluminescence and imaged with ChemiScope 5300 (Shanghai, China). The protein bands were quantified using Image J software (ImageJ 1.51j8). Three biological replicates were performed.

### LAMP2 validation using flow cytometry

To validate the relationship between LAMP2 and HIV latency, LAMP2 expression in U1 and U937 cells was detected using flow cytometry. The cells were incubated with precooled methanol for 10 min, washed twice with PBS containing 2% BSA, and then incubated for 15 min with the primary antibody against LAMP2 (ab199946, 1:200). Subsequently, they were treated with the Goat Anti-Mouse IgG H&L (Alexa Fluor 488)-conjugated secondary antibody (ab150113, 1:1000). Flow cytometric analysis was performed using a flow cytometer (Beckman, Bremen, Germany). The experiments were performed in triplicates.

### HIV-1 reactivation detection by HIV p24 expression

HIV-1 reactivation was detected using a Capsid Protein p24 ELISA kit (SEK11695, Sino Biological, Inc., Beijing, China) following the manufacturer’s protocol. Briefly, a 96-well plate with 100 µL of sample or standards in dilution buffer per well was incubated for three hours at room temperature (about 25 °C) and washed thrice with wash buffer. Subsequently, 100 µL of the detection antibody (diluted in dilution buffer to a final concentration of 0.35 µg/mL) was added to each well. The plate was then sealed and incubated for one hour at room temperature. After washing with wash buffer three times, 100 µL of the substrate solution was added to each well, incubated for 20 min at room temperature, and followed by adding 50 µL of stop solution. The optical density of each well was immediately determined using a microplate reader at 450 nm by gently tapping the plate to ensure thorough mixing.

### Statistical analysis

For proteomic data, the quantitative data file generated by the ProteinPilot software from the MS/MS intensities of the reporter tags was statistically analyzed using Microsoft Excel 2010. The relative peak intensity from U1 (114:121, 115:121, and 116:121) was compared to that from U937 (117:121, 118:121, and 119:121). DEPs were defined with a fold-change of ≥ 2 or ≤ 0.5 (*p <* 0.05) when comparing U1 to U937. For p24 quantification and western blotting, statistical analyses were performed using GraphPad Prism software (version 8.3.0). The unpaired Student’s t-test analysis was used to compare differences for two groups, while a one-way analysis of variance (ANOVA) was employed to identify significant differences among multiple groups. A statistically significant difference was considered at *p <* 0.05.

## Results

### General experiment design

**Fig. 1:**
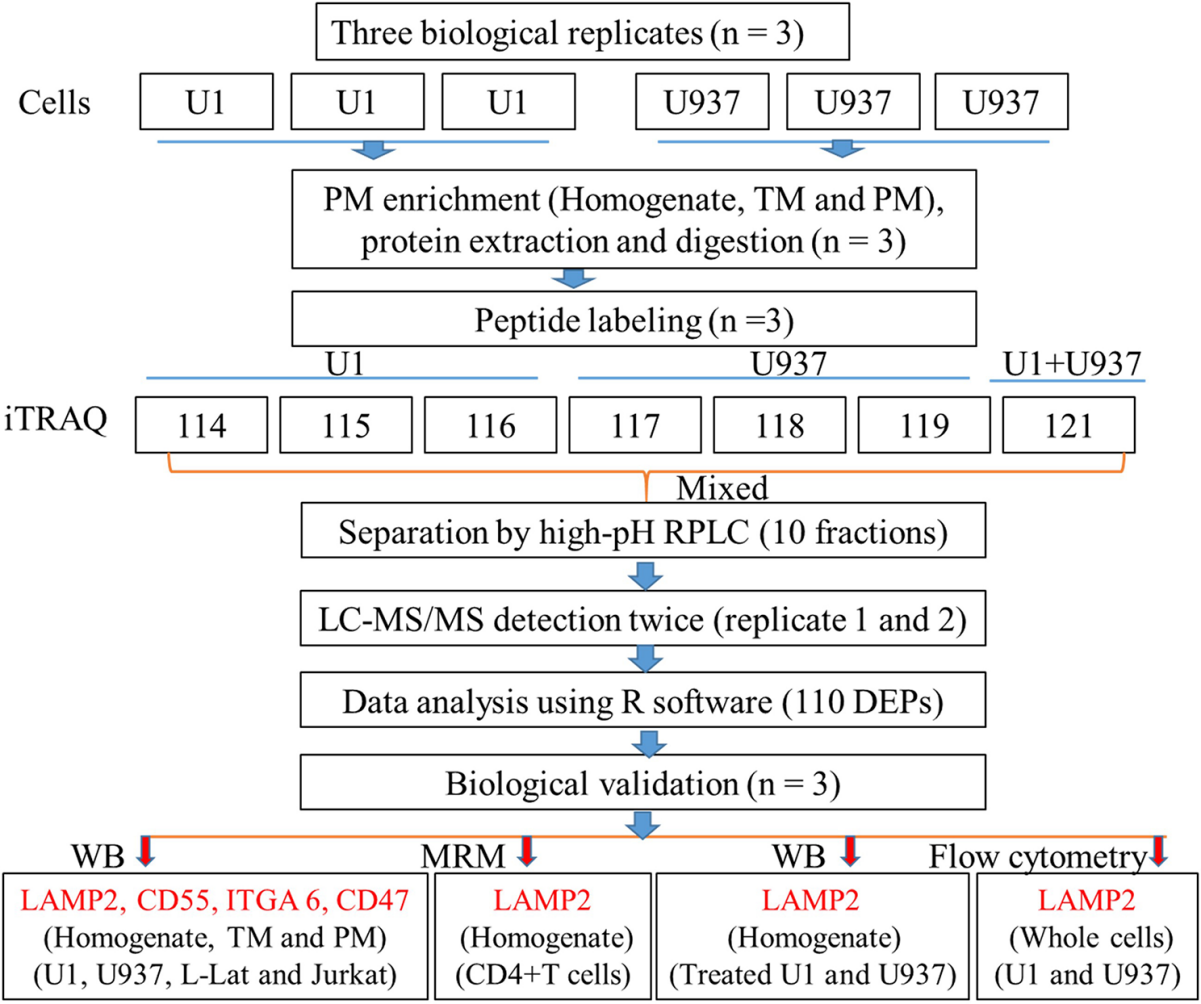
Flow chart of experiment processes Isobaric tags for relative and absolute quantitation (iTRAQ)-based quantitative proteomic analysis of U1 and U937 cell lines was performed, including three biological replicates for PM enrichment, protein extraction and digestion, and peptide labeling, and two technical replicates for LC-MS detection. PM, TM represent plasma membrane and total membrane, respectively

To discover the proteins involved in HIV latency, a PM proteomic study was performed on U1 and U937 cells in three biological replicates, including cell cultures, PM enrichment, protein extraction and digestion, and peptide labeling by iTRAQ. For protein identification by LC-MS, to decrease the possible difference produced by LC-MS procedure, two technical replicates were performed. Part of differential proteins were verified by WB, MRM, and flow cytometry in cell models and clinical samples (Fig. 1).

### PM was enriched five-fold

**Fig. 2:**
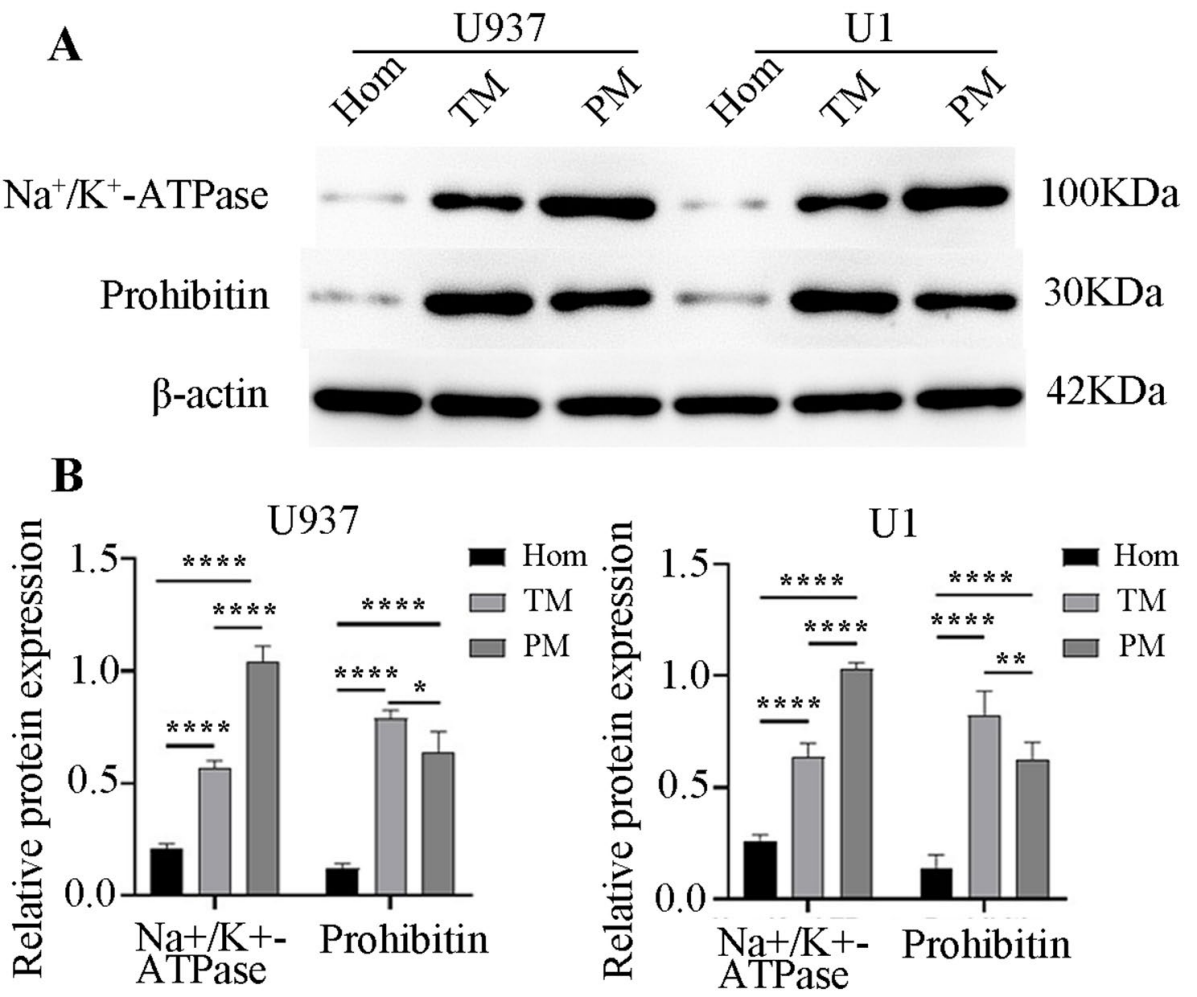
Plasma membrane (PM) enrichment efficacy detected using western blotting **A**. Detection of PM enrichment efficacy in U1 and U937 cells using western blotting. **B**. Quantification of protein bands using ImageJ software. Na^+^/K^+^-ATPase serves as a biomarker of PM, prohibitin as a biomarker of mitochondrion, and β-actin as a reference protein. Hom, TM and PM represent the homogenate of whole-cell, total membrane, and plasma membrane, respectively. The PM was enriched by more than 5- and 2-fold in the PM and TM fractions, compared with the homogenates in both U1 and U937 cells. *, **, ***, **** represent statistical significance levels of *p* < 0.05, 0.01, 0.005 and 0.001, respectively. The experiments were replicated triple

Due to their low abundance compared to many soluble proteins, PM proteins pose a challenge for studying, even with recent proteomics advances. Consequently, enriching PM is crucial for identifying these proteins in cell/tissue lysates. In this study, we utilized a PM protein extraction kit to enrich PM, evaluating membrane purification efficacy through western blotting with PM markers such as Na^+^/K^+^-ATPase and mitochondrial markers like prohibitin. As shown in Fig. 2, the PM was enriched by more than 5- and 2-fold in the PM and TM fractions, respectively, compared to the homogenates. Similar enrichment was detected in both U1 and U937 cells.

### Proteomic profiles discovered 110 DEPs

To identify PM proteins involved in HIV latency, iTRAQ-based proteomic analysis was performed using fractionated PM samples from U1 and U937 cells with twice LC-MS detections (designated as replicate 1 and 2) (Fig. 1). Analyzing the raw mass spectrometric data revealed 2,767 proteins (Table S1) and 2,748 proteins (Table S2) in the replicate 1 and 2 detection.

**Fig. 3:**
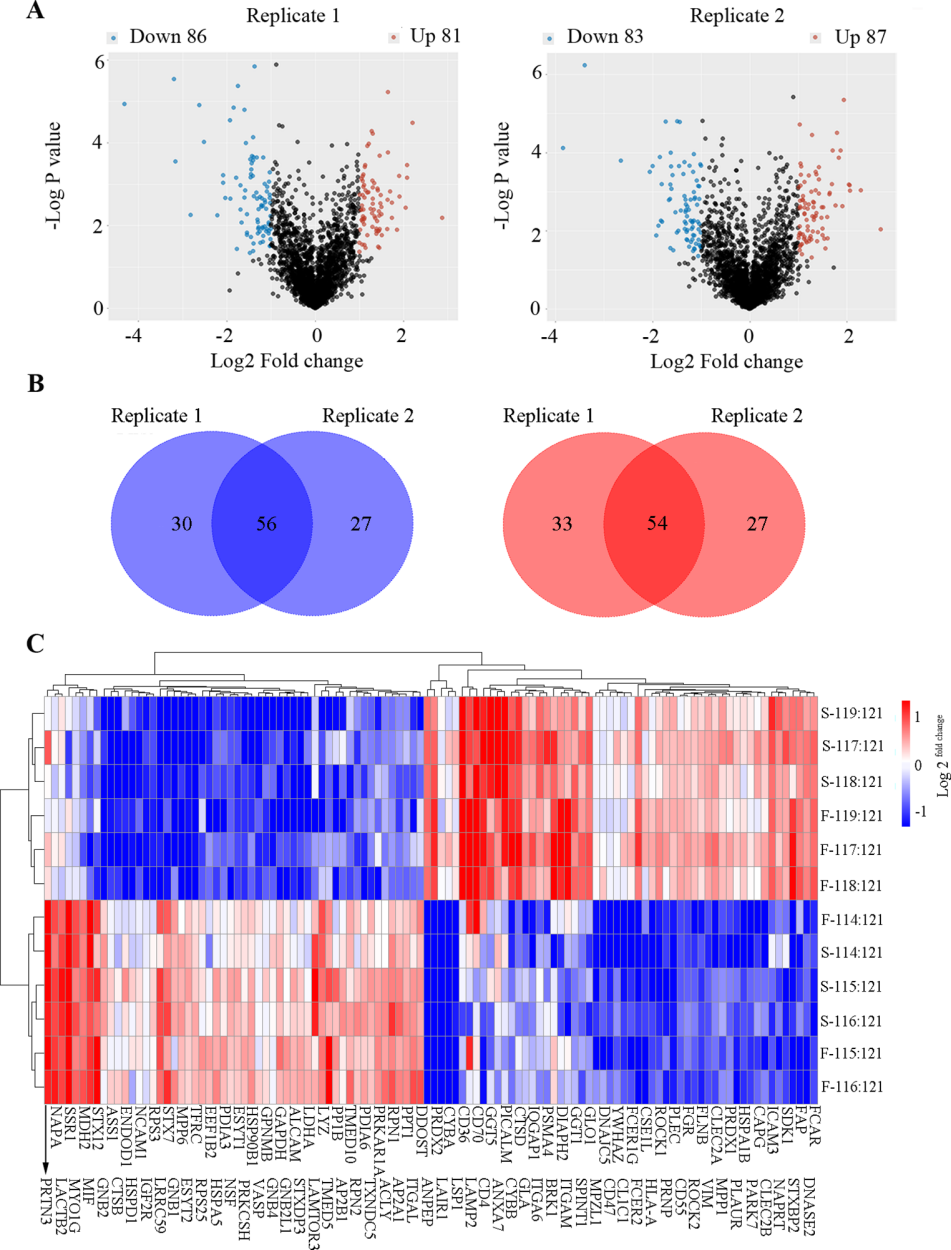
Expression analysis of the identified differential proteins (DEPs) **A**. Volcano plot showing variations in protein expression from replicates 1 (left) and 2 (right) in U1 compared to U937 cells. The fold-change and log *p-*value were inputs. The x-axis shows the average fold change (Log_2_), and the y-axis shows the negative *p-*value (-Log_10_). Compared with that in U937 cells, the upregulated proteins in U1 (fold-difference ≥ 2.0 and *p* < 0.05) are highlighted in red, downregulated proteins in U1 (fold-difference ≤ 0.5 and *p* < 0.05) in blue, and others are shown in black. **B**. Venn diagrams showing the number of DEPs in U1 compared to U937 cells in two technical replicates. Lower and higher expression levels are indicated in blue and red, respectively. **C**. Clustering of DEPs in two technical replicates using a visualized heat map. Various color intensities indicate expression levels, and a log_2_ scale was used in the color bar. The 12 labels included are indicated on the y-axis. The DEPs are shown as two lines at the bottom of the image. Odd numbers are shown in the lower line and highlighted by arrows, while even numbers are shown on the upper line. F represents replicate 1, and S represents replicate 2

Compared with U937 cells, U1 cells exhibited 86 downregulated and 81 upregulated proteins in the replicate 1 (Table S3, Fig. 3A left) and 83 downregulated and 87 upregulated proteins in the replicate 2 (Table S4, Fig. 3A right). As shown in both volcano plots (Fig. 3A), the differential proteins are mainly 2- to 4-fold difference. A total of 110 common DEPs were identified in the two replicated LC-MS analyses, including 56 downregulated and 54 upregulated proteins (Table S5 and Fig. 3B). Clustering these 110 DEPs classified the samples into two distinct clusters based on expression patterns (Fig. 3C).

#### HIV latency dysregulated the proteins involved in the immune reaction

To gain insight into the mechanism of HIV latency, we performed bioinformatics analyses of the DEPs, including BP, CC, MF and KEGG pathways. Proteins exhibiting downregulation were primarily associated with immunity processes. Notably, five of the top ten enriched biological processes were related to the immunity (Supplementary Fig. 1A and Supplementary Fig. 2A). For example, 34 proteins were enriched in the immune system process, while 24 were enriched in the leukocyte-mediated immunity process (Table S6). In terms of MF analysis, the DEPs are mainly involved in protein binding and enzyme activities (Supplementary Fig. 1B). Regarding CC, the downregulated proteins are mainly enriched in the subcellular location of PM, while upregulated proteins exhibit subcellular locations in the cytoplasm, endomembrane system and organelle membrane (Supplementary Fig. 1C). The results of the signal pathway analysis from KEGG database show that the downregulated proteins are enriched in phagosome pathway, while upregulated proteins are associate with protein processing in the endoplasmic reticulum (Supplementary Fig. 1D).

Given that latent HIV cells can evade immune recognition, we focused on proteins involved in immunity processes, which are crucial for HIV latency. We found that out of the 66 DEPs with broad immune activities, including 40 downregulated (Supplementary Fig. 2A) and 26 upregulated proteins (FDR < 0.05; Supplementary Fig. 2B; Table S7). Furthermore, as the proteins in the PM are potential targets of immune recognition, we specifically examined the cellular components of these 66 proteins, and found that 33 downregulated and 20 upregulated proteins were located in the PM (Table S7).

To determine the potential interactions of these 66 DEPs with immunity processes, a protein-protein network was constructed using the Cytoscape software. As shown in Supplementary Fig. 2C, two groups of proteins were enriched, including downregulated proteins (LAMP2, CD36, CYBB, CYBA, ITGAM, and FCAR) in the phagosomal pathway and upregulated proteins (IGF2R, AP2A1, AP2B1, and TFRC) in the endocytic pathway. Furthermore, protein-protein interactions between HIV proteins and proteins associated with immunity processes and PM location were analyzed. Fourteen proteins (such as CD4, CD55, CD36, LAMP2, and ITGAM) were found to interact with HIV proteins (Supplementary Fig. 2D and Table 1).

**Table 1:** The differential proteins regulated by HIV-1 proteins, involved in the immune system, and located in plasma membrane

Accession #	Unused	% Cov	Name	Gene name	Peptides − 95%	Fold change	*p*-value	*Regulated by HIV proteins
P63010	85.19	63.2	AP-2 complex subunit beta	AP2B1	101	2.03	1.88E-02	Tat
P16671	17.97	28.6	Platelet glycoprotein 4	CD36	21	0.2	1.62E-04	Nef/gp41
P01730	10.64	29.9	T-cell surface glycoprotein CD4	CD4	6	0.31	7.80E-03	Nef/gp120//Vpu/Vpr/gp160/gp120
P04839	21.95	34.2	Cytochrome b-245 heavy chain	CYBB	23	0.28	1.30E-03	Capsid/Tat
P06734	10.26	38.3	Low affinity immunoglobulin epsilon Fc receptor	FCER2	9	0.18	2.53E-04	gp120
P09769	18.17	38.6	Tyrosine-protein kinase Fgr	FGR	14	0.43	1.20E-03	gp120
P04439	55.19	70.7	HLA class I histocompatibility antigen, A-3 alpha chain	HLA-A	111	0.38	7.54E-04	Nef/Vpu/Tat
P10809	38.97	45.2	60 kDa heat shock protein, mitochondrial	HSPD1	24	2.32	1.55E-02	Tat
P23229	58.91	39.3	Integrin alpha-6	ITGA6	39	0.47	1.11E-03	Vif
Q6GTX8	15.93	39	Leukocyte-associated immunoglobulin-like receptor 1	LAIR1	13	0.19	2.93E-03	Nef
P13473	10.43	22	Lysosome-associated membrane glycoprotein 2	LAMP2	12	0.44	1.63E-02	Vpu/Nef
P04156	4	12.7	Major prion protein	PRNP	4	0.36	3.66E-03	Nucleocapsid
P02786	75.62	64	Transferrin receptor protein 1	TFRC	117	3.34	1.53E-04	Vpr
P08670	58.98	54.5	Vimentin	VIM	51	0.37	2.32E-04	Vpr

# from UniProtKB database

### CD55, ITGA6, CD47, and LAMP2 were confirmed to be downregulated in HIV-1-latently infected cells

**Fig. 4:**
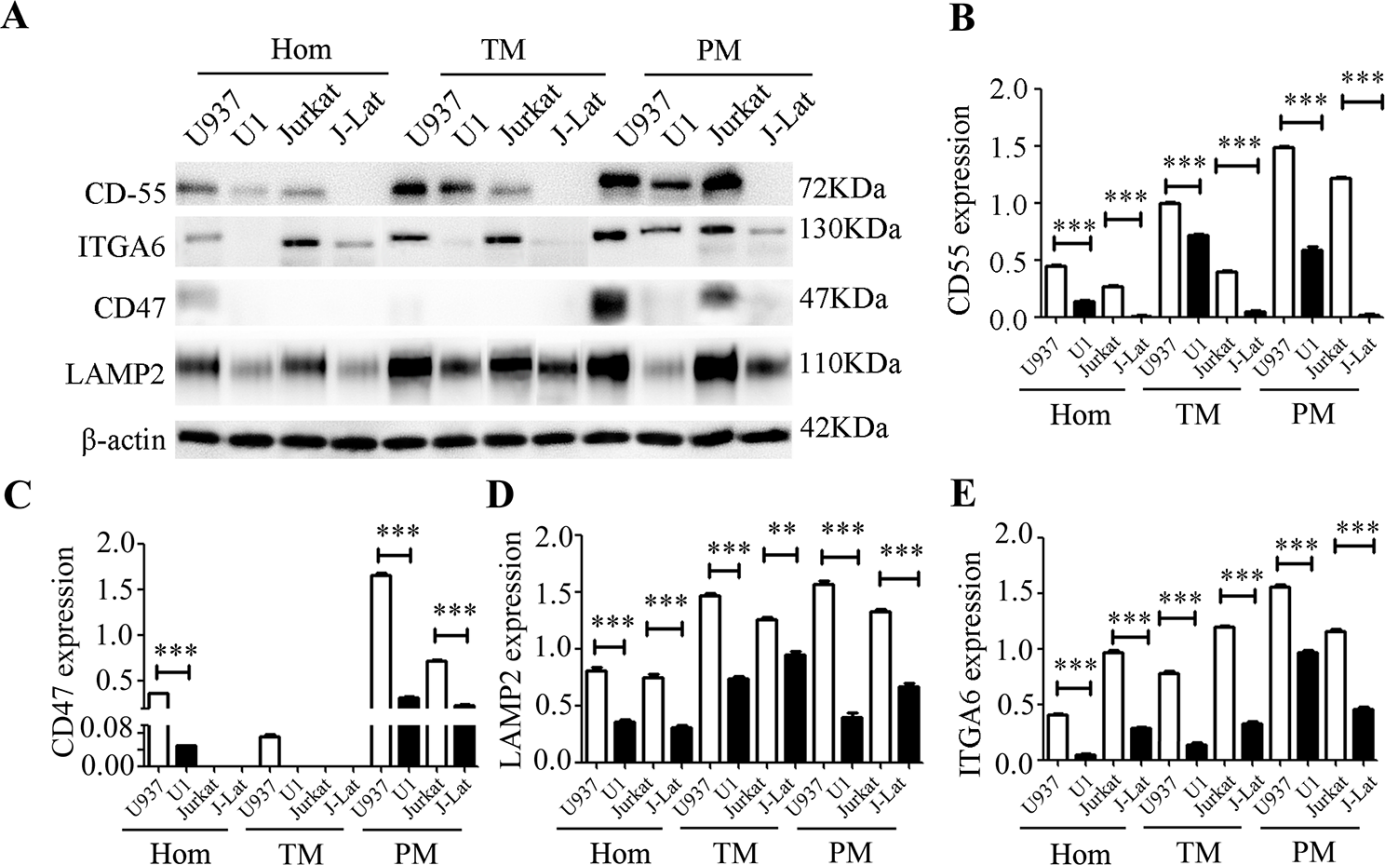
Detection of the differentially expressed proteins (DEPs) using western blotting **A**. Detection of ITGA6, CD47, CD55, LAMP2, and β-actin using western blotting in the homogenate of whole-cell (Hom), total membrane (TM), and plasma membrane (PM) from cell lines (U1, U937, J-Lat, and Jurkat). **B, C, D and E**, relative quantification of the protein bands using Image J software (ImageJ 1.51j8). β-actin was used as a reference protein. **, and *** represent statistical significance levels of *p* < 0.01, and 0.005, respectively

To confirm the DEPs related to HIV-1-latent infection, three node proteins (ITGA6, CD47, and LAMP2) and one HIV-interacting protein (CD55) were selected for western blotting. As shown in Fig. 4, all four proteins were significantly downregulated in the latent cell lines (U1 and/or J-Lat) compared with their controls (U937 and/or Jurkat). CD47 was exclusively detected in the PM fractions, whereas ITGA6, CD55, and LAMP2 were enriched in the TM and PM fractions compared with whole-cell homogenate proteins.

**Fig. 5:**
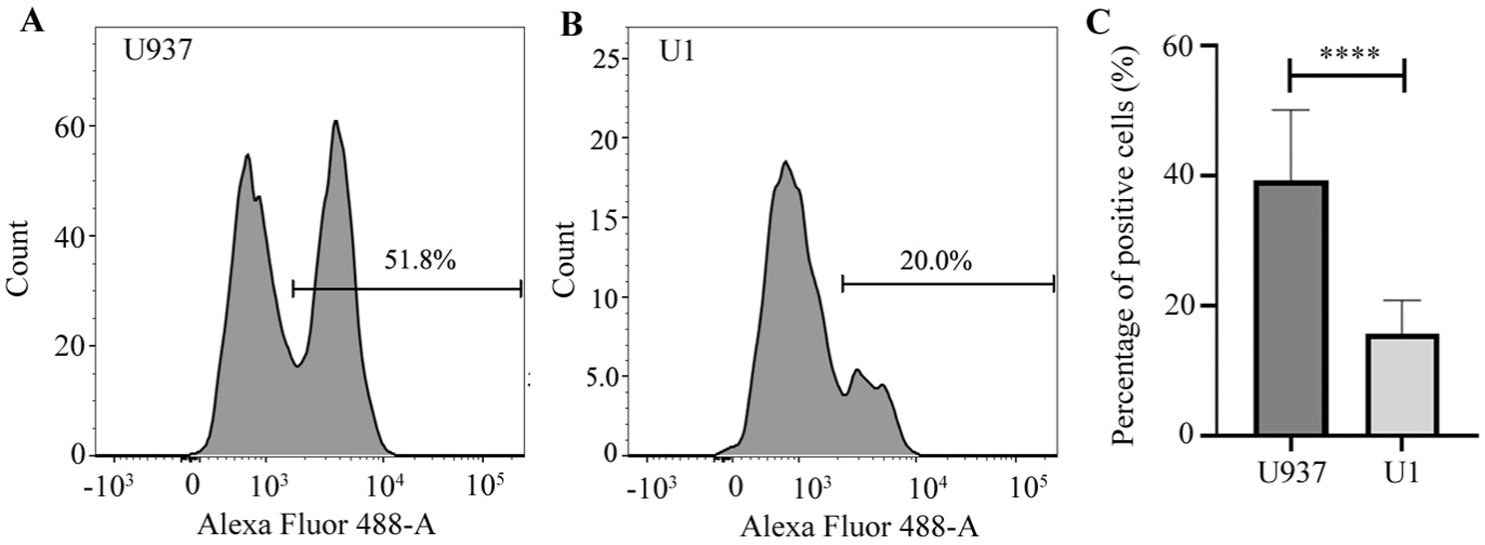
Detection of LAMP2 using flow cytometry. **A**. The profile of positive cells from U937 is depicted; **B**. the profile of positive cells from U1 is presented; **C**. The statistical results are based on three replicates (*n* = 3)

To further validate the relationship between LAMP2 and HIV-1 latency, LAMP2 expression was measured using flow cytometry. The results, presented in Fig. 5, show a significantly decrease in LAMP2 levels in U1 cells compared with U937 cells.

**Fig. 6:**
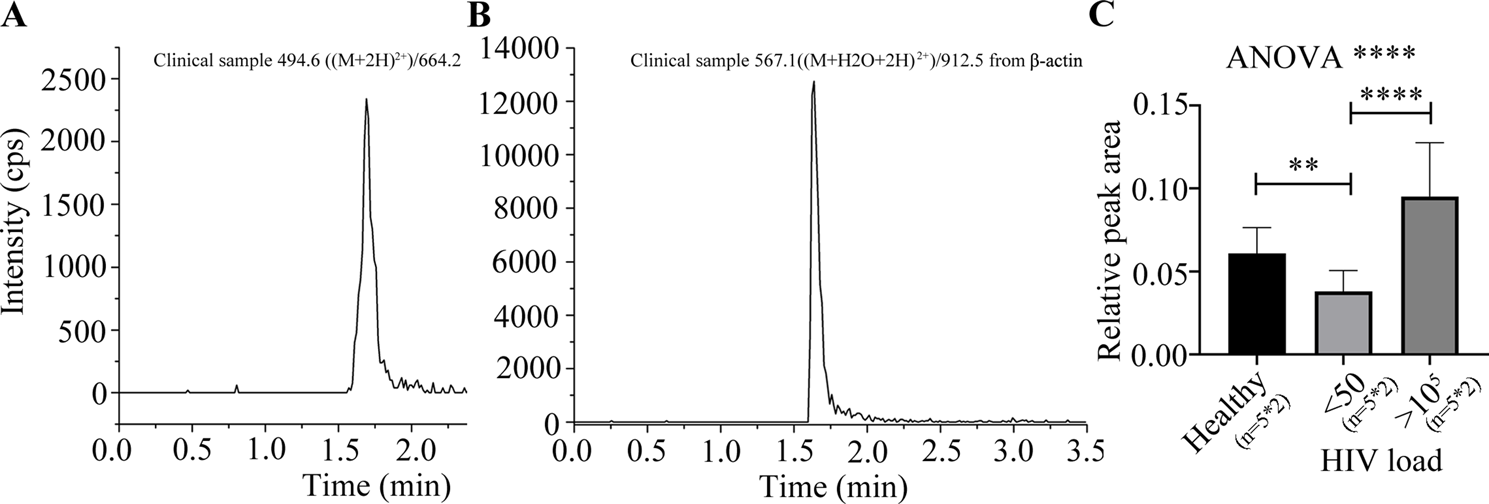
Validation of LAMP2 in resting CD4^+^ T cells using mass spectrometry-based MRM technology. **A**. LC-MS profile of the peptide from LAMP2; **B**. LC-MS profile of the peptide from β-actin; **C**. The relative peak area of LAMP2 to β-actin. Three groups were enrolled, including healthy volunteers, HIV-1 infected patients with a virus load < 50 copies/mL, and > 10^5^ copies/mL (*n* = 5/group). Each group consisted of five samples, and each sample was analyzed twice by LC-MS. T-tests, and ANOVA analysis were used for the comparison of two groups, and three groups, respectively. **, and *** represent significance levels of *p* < 0.01, and 0.005, respectively

Additionally, LAMP2 expression was validated in a patient cohort by MRM method. The results showed that LAMP2 was significantly downregulated in resting CD4^+^ T cells from HIV-1-latent patients compared with healthy volunteers. However, LAMP2 showed higher protein expression in the patients with HIV-1 virus loads higher than 10^5^ compared with that in HIV-1 latency (Fig. 6, and Table S8).

#### LAMP2 was upregulated after HIV-1 reactivation and lysosome stimulation

**Fig. 7:**
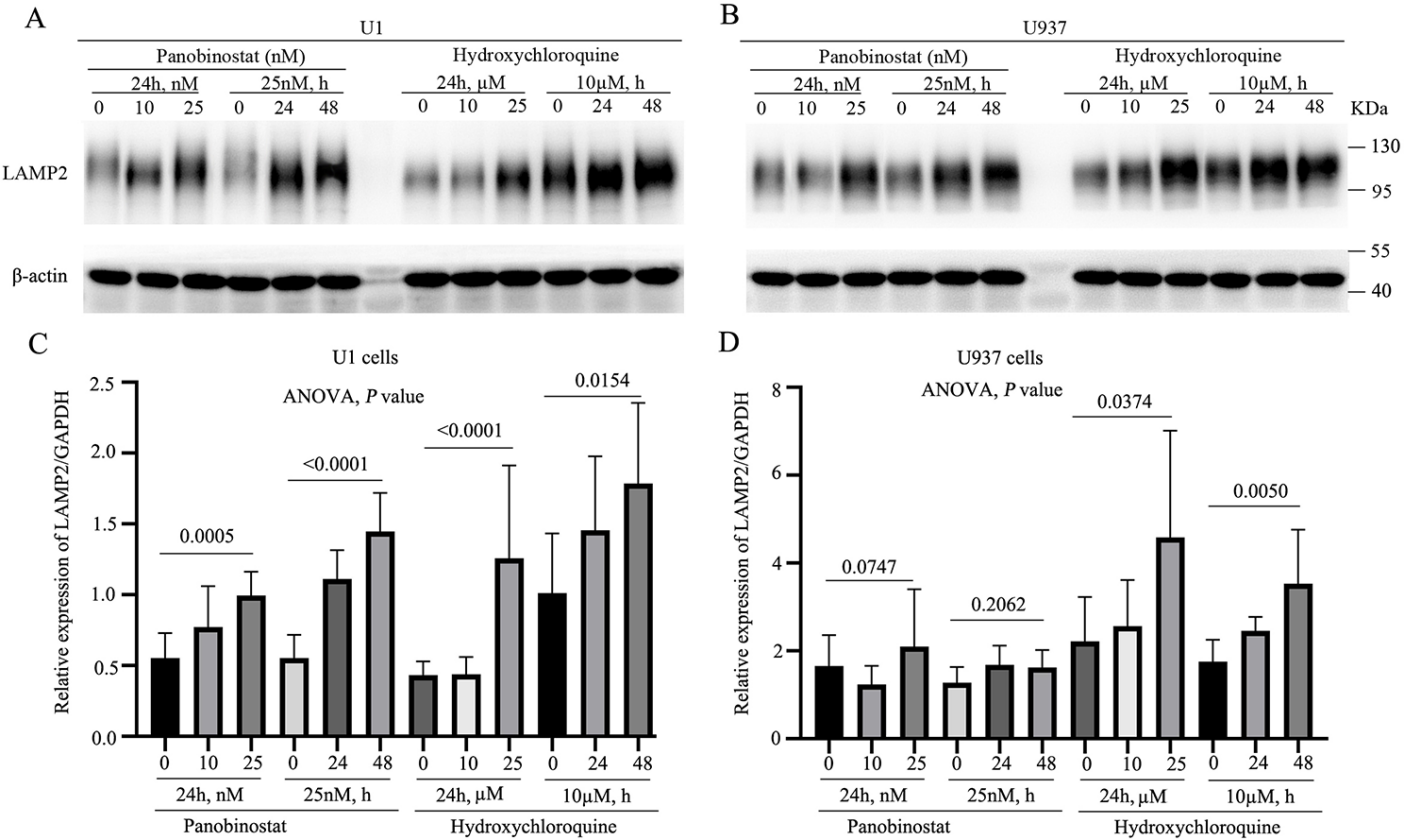
Assessment of the effect of panobinostat and hydroxychloroquine on LAMP2 protein expression. **A** and **B**. LAMP2 and β-actin were detected using western blotting using whole-cell proteins from U1 and U937 cells, respectively. **C** and **D**. Quantification of protein bands was performed using the ImageJ software. The cells were treated with panobinostat at concentrations of 0, 10, and 25 nM for 24 h (a concentration-dependent experiment), or 25 nM for 0, 24, and 48 h (a time-dependent experiment). Additionally, the cells were treated with hydroxychloroquine at concentrations of 0, 10, and 25 µM for 24 h (a concentration-dependent experiment) or 10 µM for 0, 24, and 48 h (a time-dependent experiment). Statistical analysis was performed using analysis of variance (ANOVA), and the *p* values are shown

After reactivating HIV-1 in U1 cells (Supplementary Fig. 3A and 3B), LAMP2 was found to be significantly upregulated in a concentration- and time-dependent manner in U1 cells (*p* < 0.05) (Fig. 7A and C, left). However, no significant change was detected in U937 cells (Fig. 7B, and 7D, left).

In hydroxychloroquine-treated cells, HIV-1 was slightly reactivated in U1 cells (Supplementary Fig. 3C and 3D), with no change detected in U937 cells. Notably, LAMP2 demonstrated a concentration- and time-dependent upregulation in both U1 (Fig. 7A and C, right) and U937 cells (Fig. 7B and D, right).

## Discussion

Studying HIV-1 latency faces challenges due to the lack of a suitable cell model. Resting CD4^+^ T cells [28], vital for HIV-1 cellular reservoirs investigation, are rare [29] and challenging for proteomic studies. Despite single-cell technology’s application for HIV-1 latent cells and whole-cell protein identification [10], subcellular protein identification remains difficult. This study used U1 and U937 cell lines to simulate HIV-1 latency in macrophages, conducting a subcellular proteomic analysis. DEPs were confirmed in two types of HIV-1-latent cells (U1 vs. U937, and J-Lat vs. Jurkat), as along with resting CD4^+^ T cells from patients with HIV-1 infection.

Escaping immune recognition is a significant challenge in eradicating HIV-1 from reservoirs, as latently infected cells lack viral antigen expression, evading immune detection and clearance. Proteins in the PM may offer insights into discovering latent HIV-1 infected cells due to their involvement in essential cellular functions [30, 31]. In this study, PM was enriched approximately 5-fold using a PM protein extraction kit. iTRAQ-based proteomic analysis identified over 2,700 proteins, including 110 DEPs, with more than 40% of the downregulated proteins localized in the PM (Supplementary Fig. 1).

HIV-1 alters infected cell surfaces, aiding immune evasion [32]. Therefore, identifying proteins with immune functions in the PM is crucial [33]. In this study, 66 proteins associated with immunity were identified, of which, 33 were downregulated, and 20 upregulated in U1 cells compared to U937, with cellular components in the PM. Notably, five CD proteins (CD4, CD36, CD47, CD55, and CD70) exhibited downregulation in U1 compared to U937.

Further protein-protein interaction analysis showed enrichment of downregulated proteins (such as LAMP2, CD36, and ITGAM) in phagosome pathways, while upregulated proteins were involved in the endocytosis pathway. Phagosome-lysosome fusion is critical for the intracellular killing, disrupted by HIV-1 in macrophages [34, 35]. In this study, hydroxychloroquine treatment resulted in slightly HIV-1 reactivation in U1 cells. Therefore, our study indicated that the endosomal/lysosomal pathway may be involved in HIV-1 latency.

To examine regulated proteins in HIV-host interaction, network node proteins (LAMP2, CD47, and ITGA6) and CD55 (interacting with HIV proteins) were selected and confirmed to be downregulated in HIV-1-latent cells compared with their controls. CD47 and CD55 play crucial roles in HIV-1 infection. ITGA6 can be downregulated by the varicella-zoster virus [36]. LAMP2, involved in chaperone-mediated autophagy [37] and known to regulate glucose and lipid metabolism [38], was hypothesized to be involved in HIV-1 latency through glycolipid metabolism regulation. Reactivating HIV-1 from U1 cells using panobinostat and hydroxychloroquine treatment resulted in LAMP2 upregulation, confirming its role in HIV-1 latency.

This study has a few limitations. First, LAMP2 expression was not assessed in different cell subpopulations from patient specimens. Second, the subcellular proteomic study revealed downregulation of LAMP2 in HIV-1 latent cell lines, but its expression was not detected in various subcellular organelles from clinical samples or in HIV-1 reactivated or hydroxychloroquine-treated cell models. Third, although LAMP2’s involvement in HIV-1 latency was found, its function in HIV-1 reactivation remains unexplored. Further investigations should focus on elucidating the mechanism of LAMP2 in HIV-1 latency across different cell subpopulations and subcellular organelles, as well as studying its role in HIV-1 reactivation.

## Conclusion

A subcellular proteomics study was performed and found that the downregulated proteins were mainly related to immunity in macrophage cell lines. LAMP2 was found to be downregulated in HIV-latent cells and resting CD4^+^ T cells from patients infected with HIV-1 copy number < 50, whereas, its expression was increased in patients with HIV-1 copy more than 10^5^, as well as in U1 cells after HIV-1 reactivation and lysosome regulation treated with lysosomotropic agents. Therefore, this study indicated that (1) the endosomal/lysosomal pathway was involved in HIV-1 latency, (2) LAMP2 downregulation was associated with HIV-1 latency, and (3) re-expression of LAMP2 accompanied the viral latency/productive infection transition. This study offers new clues for discovering HIV-1 latent cells and eradicating HIV-1 reservoirs. Furthermore, it is necessary to perform biological validation of LAMP2 through different cell subpopulations separated from patient specimens and to determine whether LAMP2 expression has some impact on HIV-1 reactivation through knockdown or overexpression of LAMP2 in HIV-1-latent cells.

### Electronic supplementary material

Below is the link to the electronic supplementary material.

Supplementary Material 1

Supplementary Material 2

Supplementary Material 3

Supplementary Material 4

Supplementary Material 5

Supplementary Material 6

Supplementary Material 7

Supplementary Material 8

Supplementary Material 9

Supplementary Material 10

Supplementary Material 11

Supplementary Material 12

## Data Availability

The datasets used and/or analyzed during the current study are available from. the corresponding author on reasonable request.

## Abbreviations

LAMP2: Lysosome-associated membrane glycoprotein 2
HIV: Human immunodeficiency virus
ART: Antiretroviral therapy
CD: Cluster of differentiation
PM: Plasma membrane
FDR: False discovery rate
BP: Biological process
MF: Molecular function
CC: Cellular component
DTT: Dithiothreitol
CD47: Leukocyte surface antigen CD47
ITGA6: Lntegrin alpha-6
CD4: Cluster of Differentiation 4
CD55: Complement decay-accelerating factor
CD36: Platelet glycoprotein 4
CYBB: Cytochrome b-245 heavy chain
CYBA: Cytochrome b-245 light chain
ITGAM: Integrin alpha-M
FCAR: Immunoglobulin alpha Fc receptor
IGF2R: Cation-independent mannose-6-phosphate receptor
AP2A1: AP-2 complex subunit alpha-1
AP2B1: AP-2 complex subunit beta
TFRC: Transferrin receptor protein 1
