# Supplementary figures and images for: iTRAQ-based proteomic study on monocyte cell model discovered an association of LAMP2 downregulation with HIV-1 latency
Source: Proteome Sci. 2024 May 15;22:6. doi: 10.1186/s12953-024-00230-3 (PMC11095035; doi:10.1186/s12953-024-00230-3)

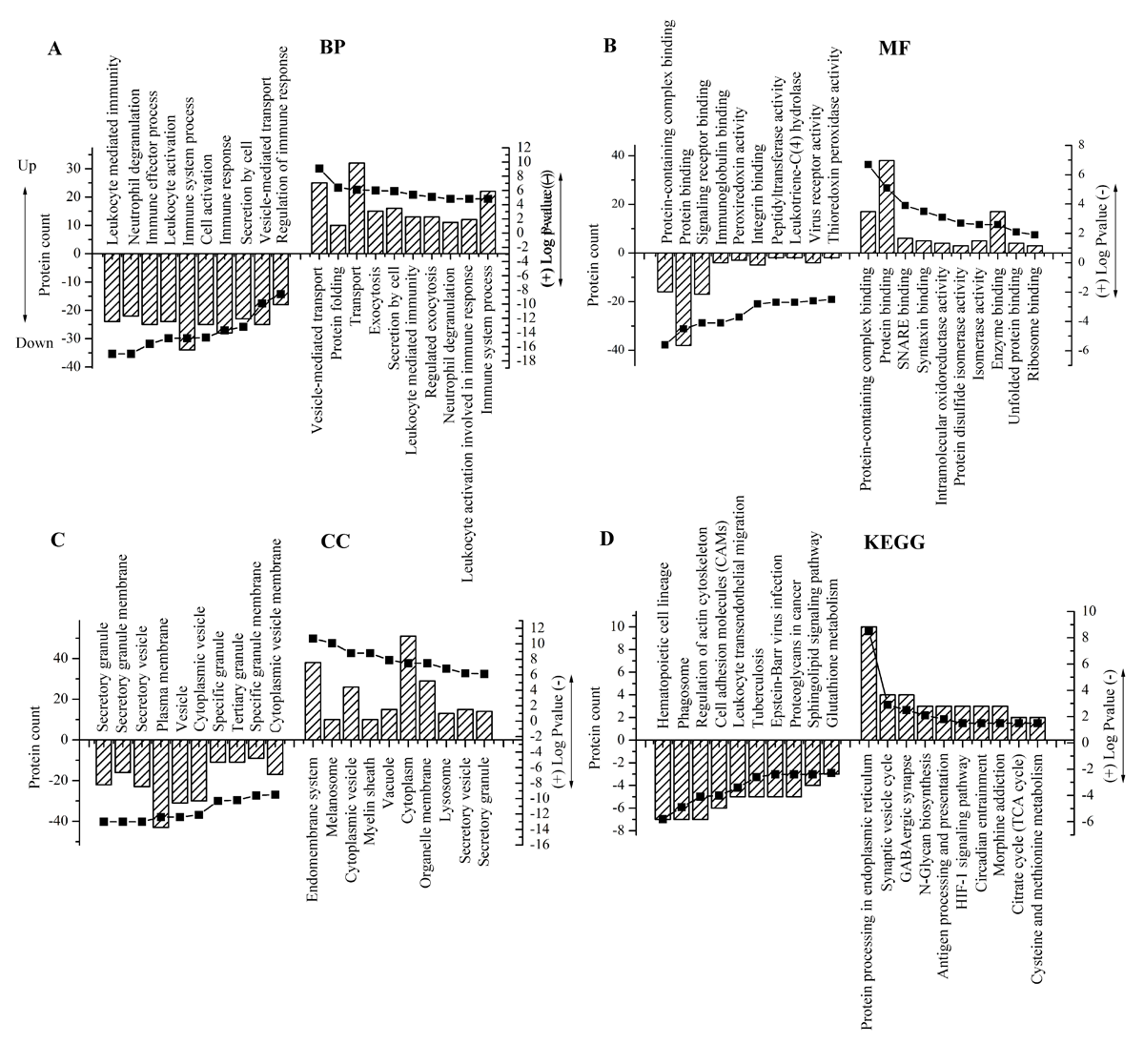

Supplement: Supplementary file 1 — Supplementary Material 1 [file 12953_2024_230_MOESM1_ESM.tif]

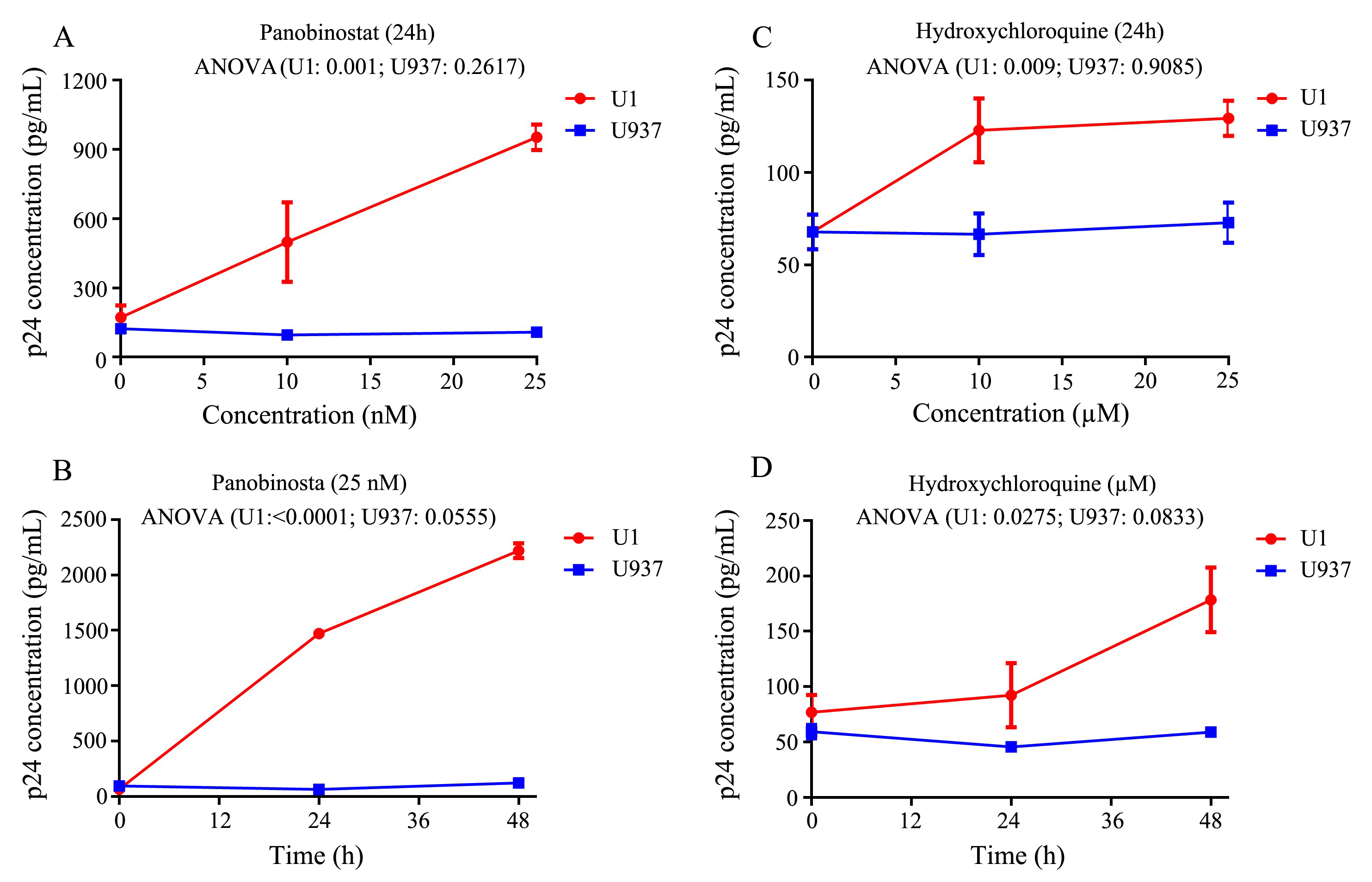

Supplement: Supplementary file 11 — Supplementary Material 11 [file 12953_2024_230_MOESM11_ESM.jpg]

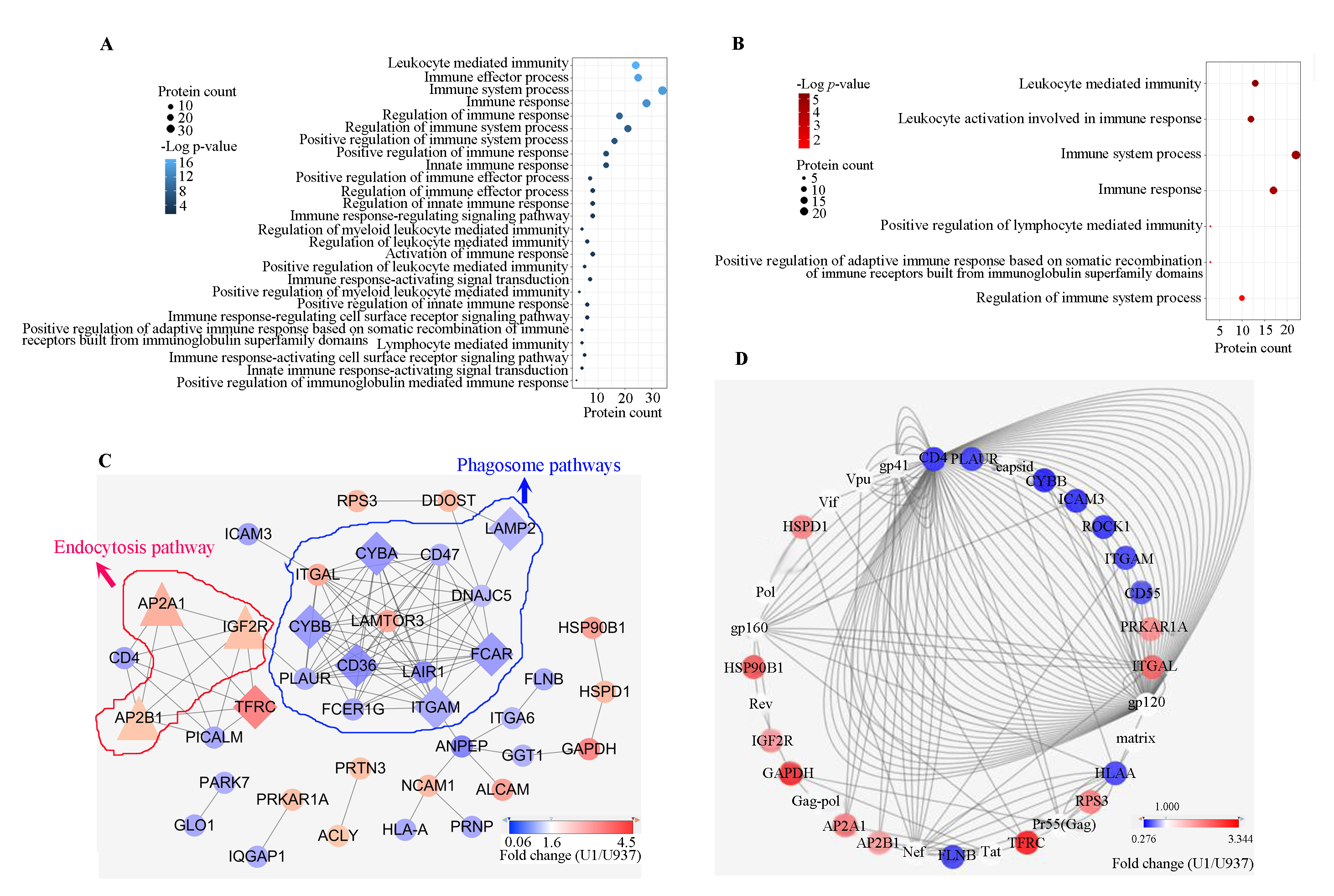

Supplement: Supplementary file 12 — Supplementary Material 12 [file 12953_2024_230_MOESM12_ESM.tif]
