# Supplementary material for: iTRAQ-based proteomic study on monocyte cell model discovered an association of LAMP2 downregulation with HIV-1 latency
Source: Proteome Sci. 2024 May 15;22:6. doi: 10.1186/s12953-024-00230-3 (PMC11095035; doi:10.1186/s12953-024-00230-3)
Supplement: Supplementary file 5 — Supplementary Material 5 [file 12953_2024_230_MOESM5_ESM.docx]

**Table S7 Differential proteins involved in the immune system**

| Accession # | Unused | % Cov | Name | Gene name | Peptides -95% | Fold change | *p*-value | Plasma membrane | *Regulated by HIV proteins |
| --- | --- | --- | --- | --- | --- | --- | --- | --- | --- |
| B0I1T2 | 81.94 | 58.9 | Unconventional myosin-Ig | MYO1G | 135 | 2.11 | 1.03E-03 | Y | NA |
| O00186 | 37.82 | 55.2 | Syntaxin-binding protein 3 | STXBP3 | 26 | 3.5 | 1.93E-03 | Y | NA |
| O15400 | 20.08 | 54.4 | Syntaxin-7 | STX7 | 24 | 4.51 | 2.33E-03 | Y | NA |
| O95782 | 101.77 | 67.9 | AP-2 complex subunit alpha-1 | AP2A1 | 95 | 2.48 | 2.40E-03 | Y | NA |
| P02786 | 75.62 | 64 | Transferrin receptor protein 1 | TFRC | 117 | 3.34 | 1.53E-04 | Y | Vpr |
| P04406 | 55.55 | 76.7 | Glyceraldehyde-3-phosphate dehydrogenase | GAPDH | 96 | 3.28 | 1.72E-03 | Y | NA |
| P10644 | 18.63 | 48.3 | cAMP-dependent protein kinase type I-alpha regulatory subunit | PRKAR1A | 18 | 2.19 | 2.54E-02 | Y | NA |
| P10809 | 38.97 | 45.2 | 60 kDa heat shock protein, mitochondrial | HSPD1 | 24 | 2.32 | 1.55E-02 | Y | Tat |
| P11717 | 105.82 | 34.7 | Cation-independent mannose-6-phosphate receptor | IGF2R | 68 | 2.14 | 2.87E-04 | Y | NA |
| P13591 | 24.35 | 26.9 | Neural cell adhesion molecule 1 | NCAM1 | 16 | 2.31 | 6.17E-03 | Y | NA |
| P14625 | 69.18 | 55.3 | Endoplasmin | HSP90B1 | 76 | 2.8 | 1.14E-03 | Y | NA |
| P20701 | 86.67 | 50.2 | Integrin alpha-L | ITGAL | 125 | 2.6 | 5.99E-03 | Y | NA |
| P23396 | 19.88 | 58.4 | 40S ribosomal protein S3 | RPS3 | 11 | 2.31 | 9.76E-04 | Y | NA |
| P24158 | 7.83 | 24.2 | Myeloblastin | PRTN3 | 7 | 2.19 | 9.56E-03 | Y | NA |
| P39656 | 21.61 | 39.3 | Dolichyl-diphosphooligosaccharide--protein glycosyltransferase 48 kDa subunit | DDOST | 17 | 2.31 | 3.00E-03 | Y | NA |
| P53396 | 60.4 | 37.8 | ATP-citrate synthase | ACLY | 36 | 2.01 | 2.06E-02 | Y | NA |
| P63010 | 85.19 | 63.2 | AP-2 complex subunit beta | AP2B1 | 101 | 2.03 | 1.88E-02 | Y | Tat |
| Q13740 | 33.6 | 47.2 | CD166 antigen | ALCAM | 38 | 2.82 | 1.02E-02 | Y | NA |
| Q14956 | 7.38 | 16.4 | Transmembrane glycoprotein NMB | GPNMB | 17 | 2.15 | 7.31E-03 | Y | NA |
| Q9UHA4 | 4.61 | 59.7 | Ragulator complex protein LAMTOR3 | LAMTOR3 | 3 | 2.59 | 7.15E-03 | Y | NA |
| O75369 | 139.13 | 48.2 | Filamin-B | FLNB | 93 | 0.38 | 3.72E-04 | Y | NA |
| P01730 | 10.64 | 29.9 | T-cell surface glycoprotein CD4 | CD4 | 6 | 0.31 | 7.80E-03 | Y | Nef/gp120//Vpu/Vpr//gp160/gp120 |
| P04156 | 4 | 12.7 | Major prion protein | PRNP | 4 | 0.36 | 3.66E-03 | Y | nucleocapsid |
| P04439 | 55.19 | 70.7 | HLA class I histocompatibility antigen, A-3 alpha chain | HLA-A | 111 | 0.38 | 7.54E-04 | Y | Nef/Vpu/Tat |
| P04839 | 21.95 | 34.2 | Cytochrome b-245 heavy chain | CYBB | 23 | 0.28 | 1.30E-03 | Y | Capsid/Tat |
| P06734 | 10.26 | 38.3 | Low affinity immunoglobulin epsilon Fc receptor | FCER2 | 9 | 0.18 | 2.53E-04 | Y | gp120 |
| P08174 | 19.43 | 49.6 | Complement decay-accelerating factor | CD55 | 18 | 0.43 | 2.92E-03 | Y | NA |
| P08670 | 58.98 | 54.5 | Vimentin | VIM | 51 | 0.37 | 2.32E-04 | Y | Vpr |
| P09769 | 18.17 | 38.6 | Tyrosine-protein kinase Fgr | FGR | 14 | 0.43 | 1.20E-03 | Y | gp120 |
| P11215 | 42.12 | 31.7 | Integrin alpha-M | ITGAM | 28 | 0.39 | 1.63E-03 | Y | NA |
| P13473 | 10.43 | 22 | Lysosome-associated membrane glycoprotein 2 | LAMP2 | 12 | 0.44 | 1.63E-02 | Y | Vpu/Nef |
| P13498 | 8.2 | 38.5 | Cytochrome b-245 light chain | CYBA | 13 | 0.26 | 4.82E-03 | Y | NA |
| P15144 | 89.13 | 55.6 | Aminopeptidase N | ANPEP | 128 | 0.1 | 1.74E-06 | Y | NA |
| P16671 | 17.97 | 28.6 | Platelet glycoprotein 4 | CD36 | 21 | 0.2 | 1.62E-04 | Y | Nef/gp41 |
| P19440 | 36.84 | 41.3 | Gamma-glutamyltranspeptidase 1 | GGT1 | 41 | 0.43 | 2.43E-03 | Y | NA |
| P23229 | 58.91 | 39.3 | Integrin alpha-6 | ITGA6 | 39 | 0.47 | 1.11E-03 | Y | Vif |
| P24071 | 8.04 | 28.2 | Immunoglobulin alpha Fc receptor | FCAR | 7 | 0.28 | 7.25E-05 | Y | NA |
| P30273 | 8 | 33.7 | High affinity immunoglobulin epsilon receptor subunit gamma | FCER1G | 4 | 0.36 | 1.04E-02 | Y | NA |
| P32942 | 7.77 | 16.8 | Intercellular adhesion molecule 3 | ICAM3 | 7 | 0.32 | 1.71E-03 | Y | NA |
| P32970 | 3.89 | 16.1 | CD70 antigen | CD70 | 2 | 0.32 | 4.80E-03 | Y | NA |
| P46940 | 178.77 | 61 | Ras GTPase-activating-like protein IQGAP1 | IQGAP1 | 242 | 0.4 | 7.09E-03 | Y | NA |
| Q00013 | 16.34 | 31.8 | 55 kDa erythrocyte membrane protein | MPP1 | 14 | 0.45 | 3.37E-03 | Y | NA |
| Q03405 | 22.32 | 45.7 | Urokinase plasminogen activator surface receptor | PLAUR | 32 | 0.39 | 1.12E-03 | Y | NA |
| Q04760 | 24.17 | 76.1 | Lactoylglutathione lyase | GLO1 | 31 | 0.36 | 4.45E-04 | Y | NA |
| Q08722 | 11.27 | 17 | Leukocyte surface antigen CD47 | CD47 | 13 | 0.43 | 8.20E-03 | Y | NA |
| Q13464 | 62.2 | 40.3 | Rho-associated protein kinase 1 | ROCK1 | 54 | 0.33 | 8.29E-04 | Y | NA |
| Q13492 | 5.79 | 11.8 | Phosphatidylinositol-binding clathrin assembly protein | PICALM | 3 | 0.35 | 1.42E-02 | Y | NA |
| Q15833 | 6.19 | 15.2 | Syntaxin-binding protein 2 | STXBP2 | 6 | 0.23 | 2.83E-03 | Y | NA |
| Q6GTX8 | 15.93 | 39 | Leukocyte-associated immunoglobulin-like receptor 1 | LAIR1 | 13 | 0.19 | 2.93E-03 | Y | Nef |
| Q6UVW9 | 8.95 | 37.9 | C-type lectin domain family 2 member A | CLEC2A | 11 | 0.37 | 1.91E-03 | Y | NA |
| Q92478 | 13 | 61.7 | C-type lectin domain family 2 member B | CLEC2B | 29 | 0.38 | 4.12E-03 | Y | NA |
| Q99497 | 21.26 | 83.1 | Protein deglycase DJ-1 | PARK7 | 25 | 0.42 | 1.92E-03 | Y | NA |
| Q9H3Z4 | 5.76 | 48 | DnaJ homolog subfamily C member 5 | DNAJC5 | 7 | 0.49 | 2.11E-02 | Y | NA |
| P00966 | 7.68 | 24.8 | Argininosuccinate synthase | ASS1 | 5 | 3.33 | 1.95E-03 | N | NA |
| O00115 | 7.34 | 18.3 | Deoxyribonuclease-2-alpha | DNASE2 | 7 | 0.31 | 1.25E-04 | N | NA |
| P06280 | 18.74 | 37.5 | Alpha-galactosidase A | GLA | 13 | 0.46 | 7.69E-03 | N | NA |
| P07339 | 41.46 | 58.7 | Cathepsin D | CTSD | 123 | 0.33 | 1.17E-03 | N | NA |
| P07858 | 17.52 | 37.8 | Cathepsin B | CTSB | 15 | 2.74 | 9.20E-03 | N | NA |
| P0DMV9 | 41.02 | 73.5 | Heat shock 70 kDa protein 1B | HSPA1B | 71 | 0.4 | 3.42E-03 | N | NA |
| P14174 | 5.08 | 35.7 | Macrophage migration inhibitory factor | MIF | 10 | 3.82 | 1.29E-03 | N | NA |
| P30101 | 73.12 | 74.1 | Protein disulfide-isomerase A3 | PDIA3 | 77 | 2.29 | 6.27E-03 | N | NA |
| P61626 | 21.43 | 61.5 | Lysozyme C | LYZ | 24 | 3.49 | 2.13E-03 | N | NA |
| Q06830 | 30.89 | 74.4 | Peroxiredoxin-1 | PRDX1 | 32 | 0.44 | 9.53E-03 | N | NA |
| Q6XQN6 | 19.91 | 39 | Nicotinate phosphoribosyltransferase | NAPRT | 13 | 0.4 | 3.10E-03 | N | NA |
| Q8NBS9 | 32.67 | 56.3 | Thioredoxin domain-containing protein 5 | TXNDC5 | 32 | 2.16 | 1.93E-03 | N | NA |
| Q8WUW1 | 6.94 | 57.3 | Protein BRICK1 | BRK1 | 5 | 0.43 | 2.19E-02 | N | NA |

# from UniProtKB database.

*NA, no HIV regulation information, or inconsistent regulation is shown.
