# Supplementary material for: iTRAQ-based proteomic study on monocyte cell model discovered an association of LAMP2 downregulation with HIV-1 latency
Source: Proteome Sci. 2024 May 15;22:6. doi: 10.1186/s12953-024-00230-3 (PMC11095035; doi:10.1186/s12953-024-00230-3)

**All original, full-length gels and blot images**

**Fig. 1. Plasma membrane (PM) enrichment detected using western blotting**

**The primary images for Figure 1**

**1) Na^+^-K^+^-ATPase**: from left to right, the protein lanes are marker, and homogenate, total membrane, and plasma membrane from U937 cells, and U1 cells, respectively.

Replicate 1


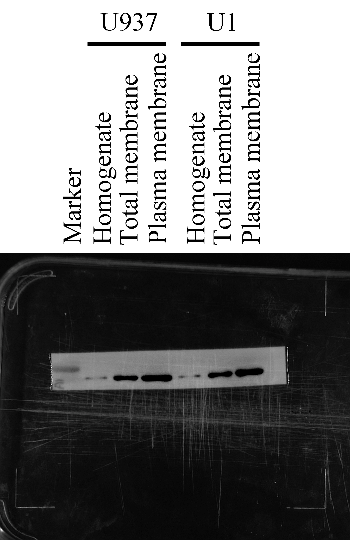


Replicate 2


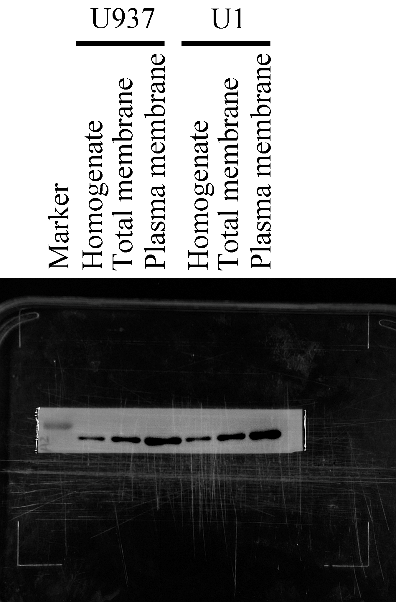


Replicate 3


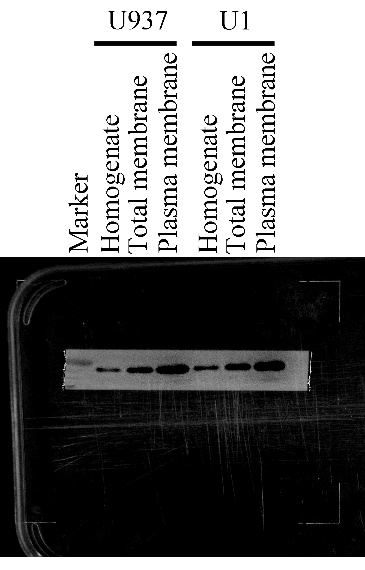


**2) Prohibitin**: from left to right, the protein lanes are marker, and homogenate, total membrane, and plasma membrane from U937 cells, and U1 cells, respectively.

Replicate 1


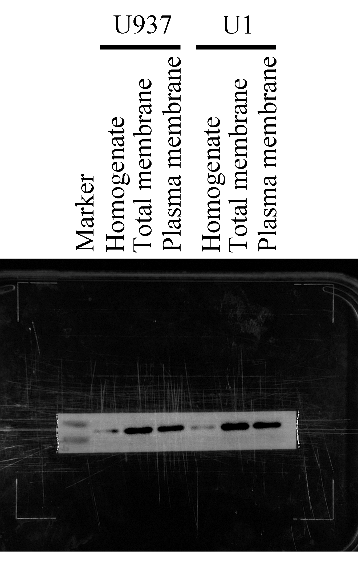


Replicate 2


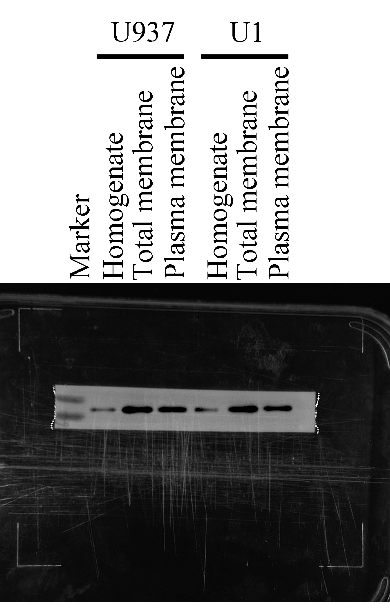


Replicate 3


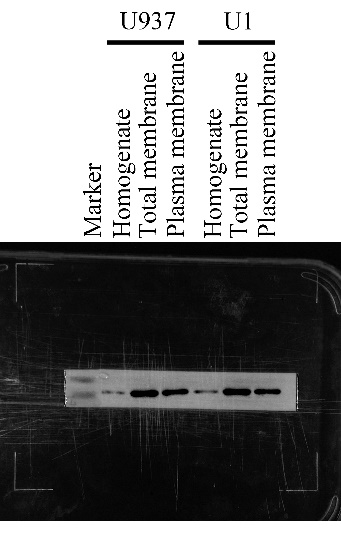


**3) β-Actin**: from left to right, the protein lanes are marker, and homogenate, total membrane, and plasma membrane from U937 cells, and U1 cells, respectively.

Replicate 1


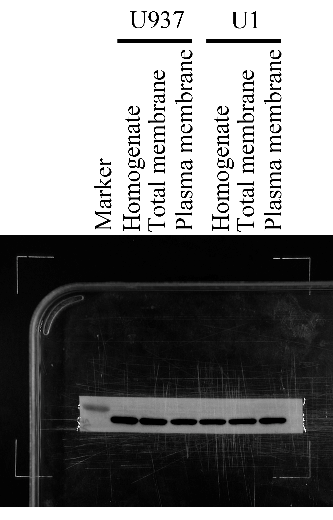


Replicate 2


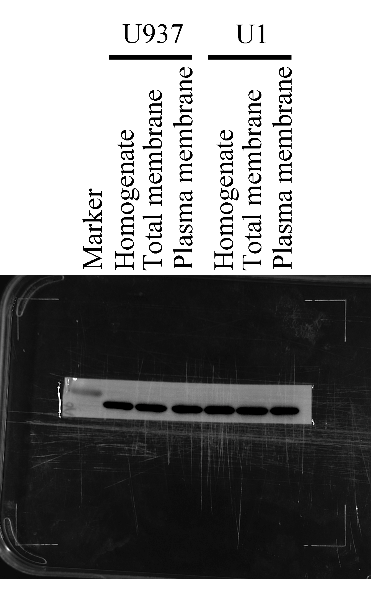


Replicate 3


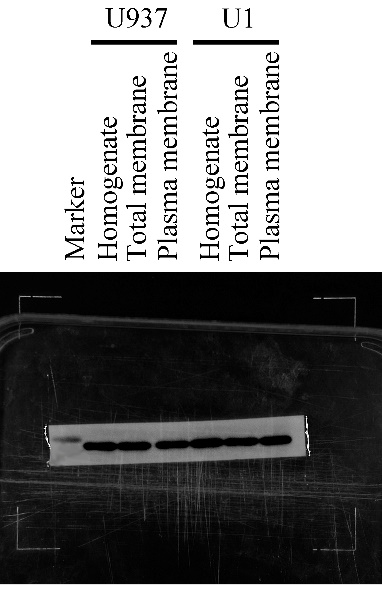


**Fig. 4. Detection of the differentially expressed proteins using western blotting**

**The primary images for Figure 4**

Positive was a mix from HepG2, U87, MCF-7 and Hela cells.

Total, TM and PM were protein samples from homogenate, total membrane and plasma membrane, respectively. Comment: In replicate 1, the markers were not completely saved, therefore, we cannot label them now.

**CD55**

Replicate 1


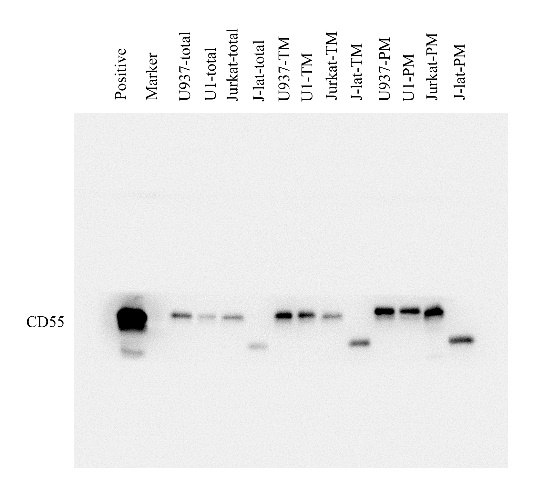


Replicate 2


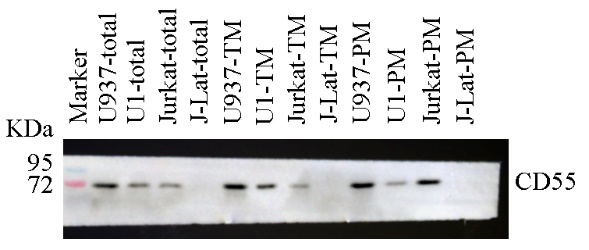


Replicate 3


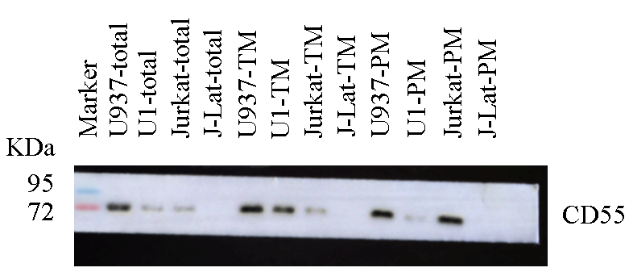


**CD47**

**
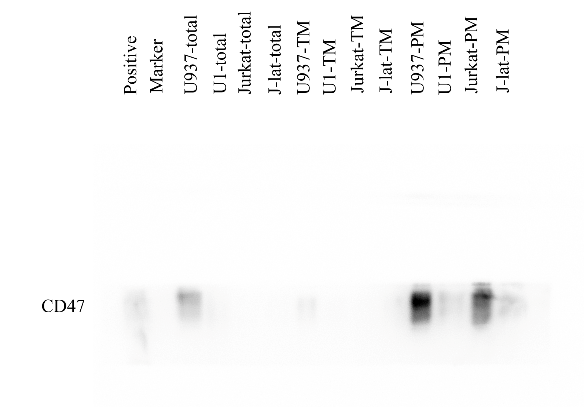
**

**Replicate 2**

**
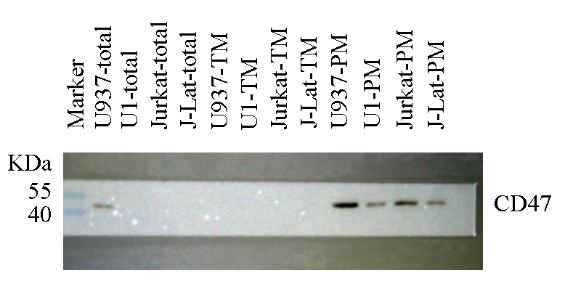
**

**Replicate 3**

**
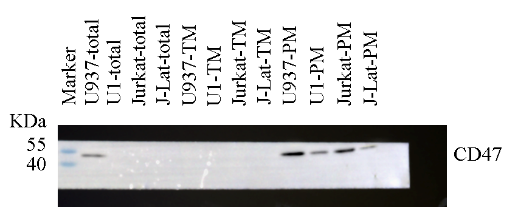
**

**ITGA6**

**
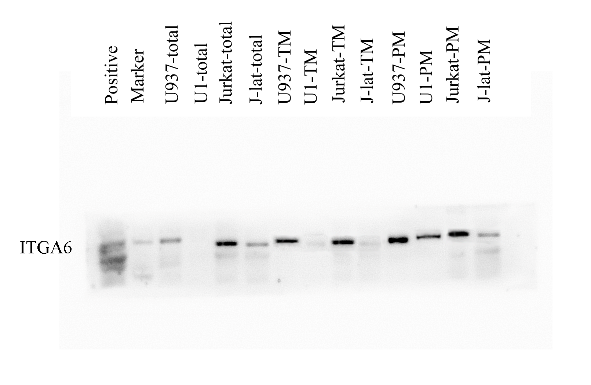
**

**Replicate 2**

**
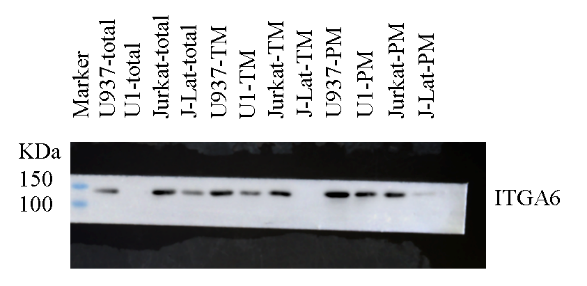
**

**Replicate 3**

**
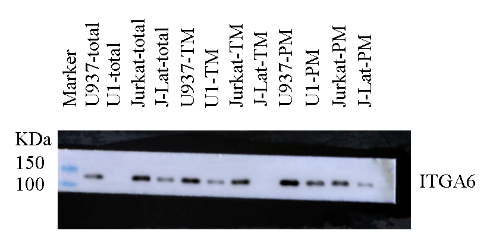
**

**LAMP2**

**Replicate 1**

**
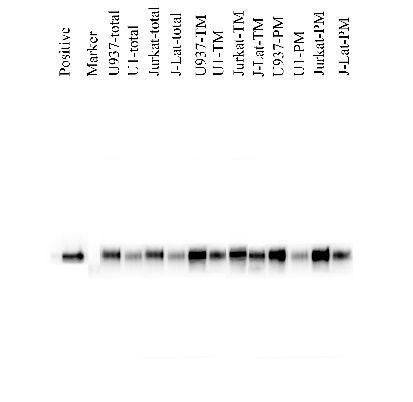
**

**Replicate 2**

**
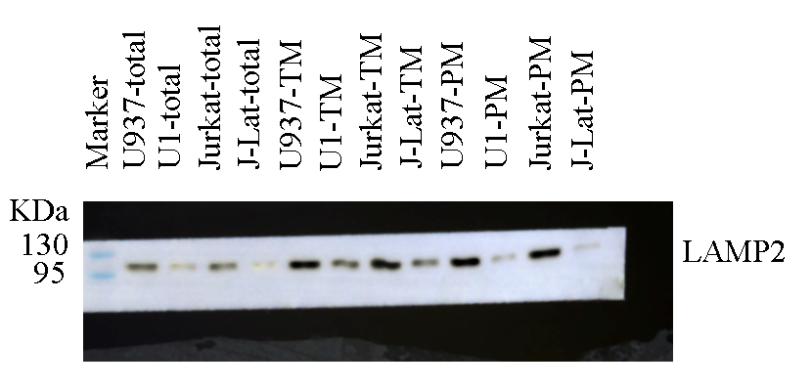
**

**Replicate 3**

**
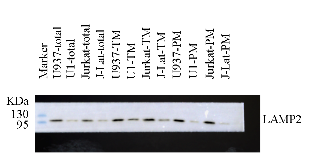
**

**β-actin**

**
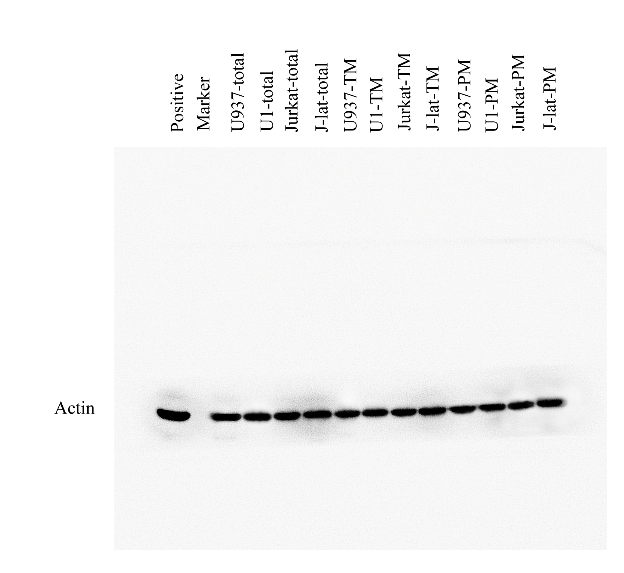
**

**Replicate 2**

**
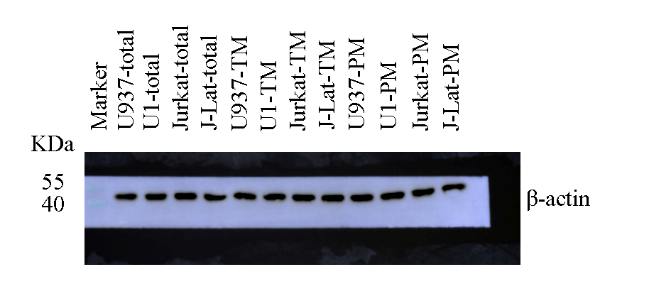
**

**Replicate 3**

**
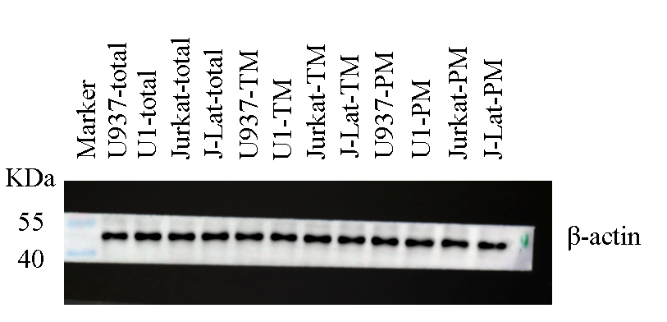
**

**Fig. 7. Assessment of the effect of panobinostat and hydroxychloroquine on LAMP2 protein expression**

**The primary images with three replicates for Figure 7**

**A. In U937 and U1 cells**

The LAMP2 protein level after panobinostat and hydroxychloroquine treatment in replicate 1 and 2. P and H represent panobinostat and hydroxychloroquine respectively. Comment: the protein levels in U1 are too low to distinguish the difference, therefore we re-examed LAMP2 in U1 cells.

**Time-dependence of LAMP2 only used for U937 analysis.**

Replicate 1

**
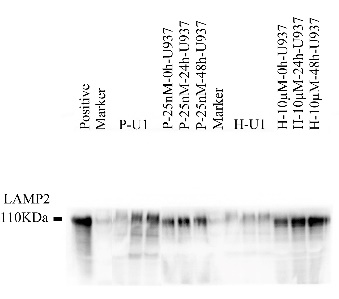
**

**Replicate 2**

**
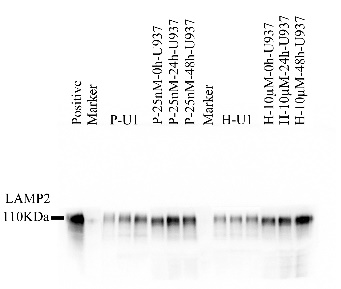
**

**Concentration-dependence of LAMP2 only used for U937 analysis**

**Replicate 1**

**
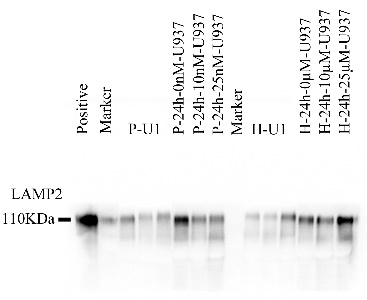

Replicate 2**

**
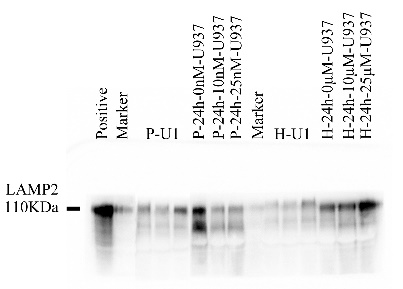
**

**GAPDH**

**Time-dependence for GAPDH for LAMP2 analysis**

**Replicate 1**


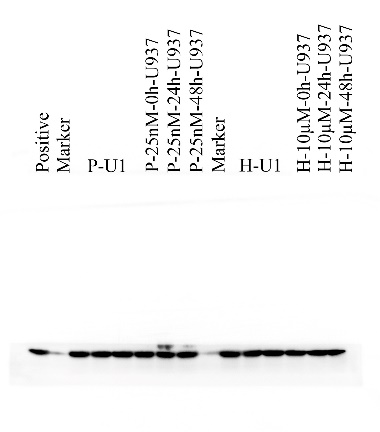


Replicate 2


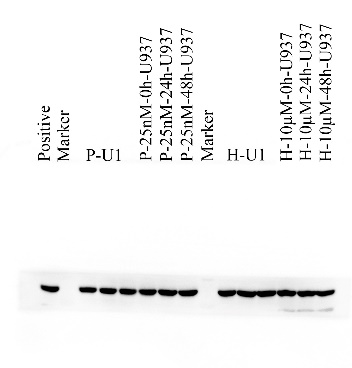

**Concentration-dependence for GAPDH for LAMP2 analysis**

**Replicate 1**

**
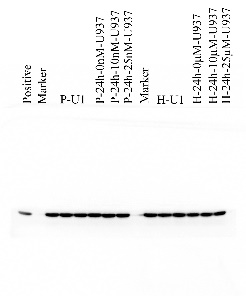
**

**Replicate 2**

**
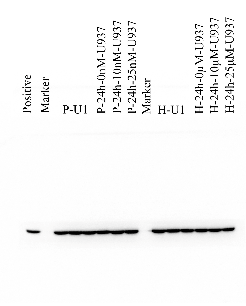
**

**B. U1 cells**

**Concentration and time-dependence of LAMP2 in U1 cells**

**LAMP2**

**Replicate 1**

**
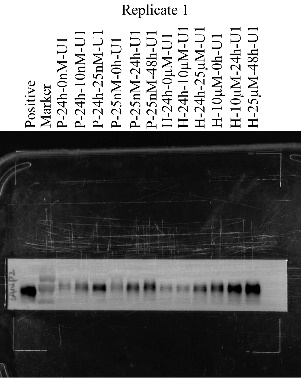
**

**Replicate 2**

**
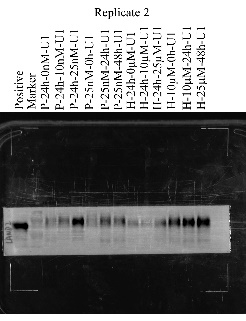
**

**GAPDH**

**Replicate 1**

**
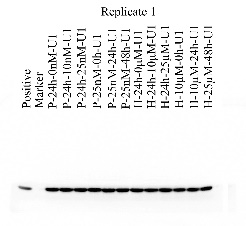
**

**Replicate 2**

**
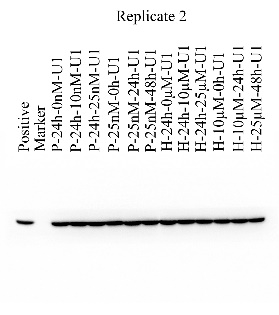
**

**C. In U937 or U1 cells**

To clearly show the time-dependence and concentration-dependence in U937 and U1 for panobinostat (P) or hydroxychloroquine (H) treatment, all kinds of proteins from U1 or U937 were singly loaded in one gel in the replicate 3.

**LAMP2**

In U937 cells


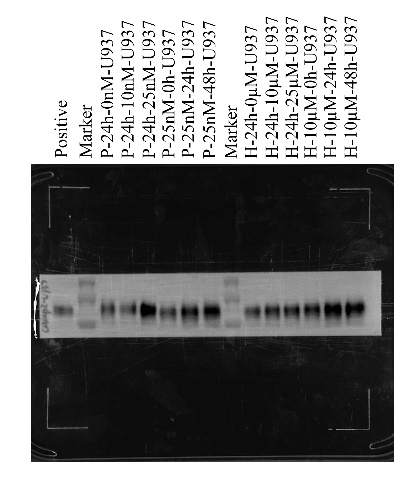


In U1 cells


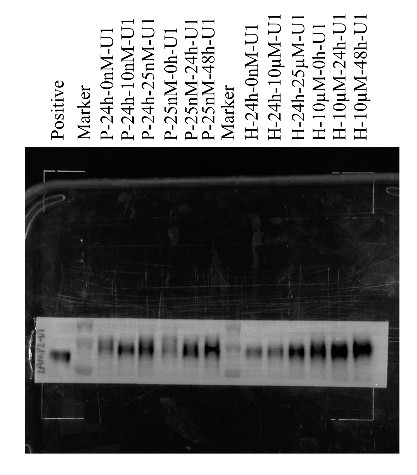


β-actin

In U937


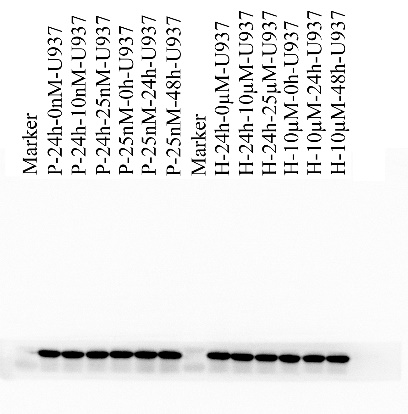


In U1 cells


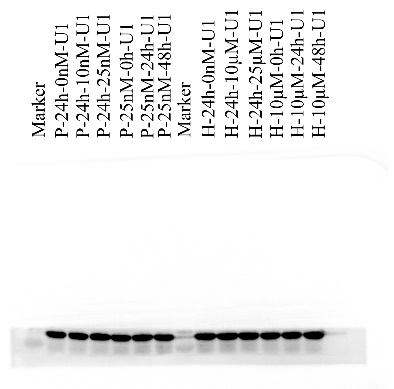

Supplement: Supplementary file 7 — Supplementary Material 7 [file 12953_2024_230_MOESM7_ESM.docx]
